# Behaviourally informed text message reminders to increase cervical screening attendance in people with severe mental illness: the OPTIMISE pilot randomised controlled trial

**DOI:** 10.1136/bmjopen-2026-122934

**Published:** 2026-09-11

**Authors:** Beth Nichol, Andrew Bryant, Lesley Hall, Christian von Wagner, Elizabeth Barley, Aikaterini Grimani, Emily Oliver, David Osborn, Luke Vale, Ivo Vlaev, Fiona Graham

**Affiliations:** 1 NIHR Policy Research Unit Behavioural and Social Sciences, Newcastle University, Newcastle upon Tyne, UK; 2 School of Communities and Education, Northumbria University, Newcastle upon Tyne, UK; 3 Newcastle University Population Health Sciences Institute, Newcastle upon Tyne, UK; 4 Behavioural Science and Health, University College London, London, UK; 5 School of Health Sciences, University of Surrey, Guildford, UK; 6 NIHR Policy Research Unit Behavioural and Social Sciences, University of Warwick, Coventry, UK; 7 Division of Psychiatry, University College London, London, UK; 8 NIHR Policy Research Unit in Mental Health, University College London, London, UK; 9 NIHR Policy Research Unit Behavioural and Social Sciences, London School of Hygiene and Tropical Medicine, London, UK; 10 NIHR Policy Research Unit Behavioural and Social Sciences, National University of Singapore, Singapore

**Keywords:** PUBLIC HEALTH, MENTAL HEALTH, Behavior

## Abstract

**Objectives:**

This study assessed the feasibility of both the delivery and evaluation of ‘enhanced’ (behaviourally informed) text message reminders containing links to existing co-designed resources supporting decision-making for people with severe mental illness (SMI) regarding attendance of cervical screening.

**Design:**

A pilot randomised controlled trial (RCT).

**Setting:**

13 General Practice (GP) practices in London were recruited.

**Participants:**

GP practices identified people with SMI aged 24–64 years who were overdue cervical screening. Target sample size was 120 participants (60 per arm) based on existing guidance for pilot trials.

**Intervention:**

In March 2025, participants were randomised (1:1) to receive either the enhanced (intervention) or the standard (control) SMS reminder.

**Primary and secondary outcome measures:**

18 weeks later, feasibility outcomes were collected (primary outcomes) and data analysis for a definitive RCT was rehearsed (secondary outcome).

**Results:**

Of the 150 participants across 13 GP practices that were randomised (n=75 per arm), 132 (88%) texts delivered (intervention n=64/75 (85%), control n=68/75 (91%)). 10 practices (76.9%) provided follow-up data for 102 participants (intervention n=50, control n=52). Five participants (intervention n=4, control n=1) attended screening within the trial period. Participant survey response rate was low (9/132 (7%), intervention n=5, control n=4). Both SMS messages were low cost, with the intervention SMS a 50% higher cost to deliver (7.5p vs 5p per SMS). Primary feasibility measures of recruitment rate of GP practices (27%), retention of GP practices (77%) and participants (100%), SMS delivery (88%) and data completeness (64%) indicated viability, although survey response rate (7%) did not.

**Conclusions:**

Achieving adequate recruitment and retention, data completeness and comparable groups is viable with some amendments, although an alternative method is required to assess fidelity. Behaviourally informed SMS reminders are feasible to deliver to people with SMI, although it is uncertain if the extra resources are accessed and used. With changes to data collection, a definitive trial could be feasible. Given the low observed cervical screening attendance, additional intervention is needed for this group.

**Trial registration number:**

ISRCTN12558681.

**STRENGTHS AND LIMITATIONS OF THIS STUDY**

- This study successfully recruited and retained participants with severe mental illness who had not responded to previous cervical screening invitations.
- Limitations in the accuracy and completeness of data extracted from general practice records (particularly around previous screening attendance) requires rectification for a fully powered trial.
- Difficulties experienced by participating GP practices in extracting outcome data may have introduced reporting errors.
- An intention-to-treat analysis was not possible due to system limitations by the text message provider.
- Prespecified progression criteria were not defined, limiting objective assessment of the feasibility of a definitive trial.

## Introduction/background

**Figure 1:**
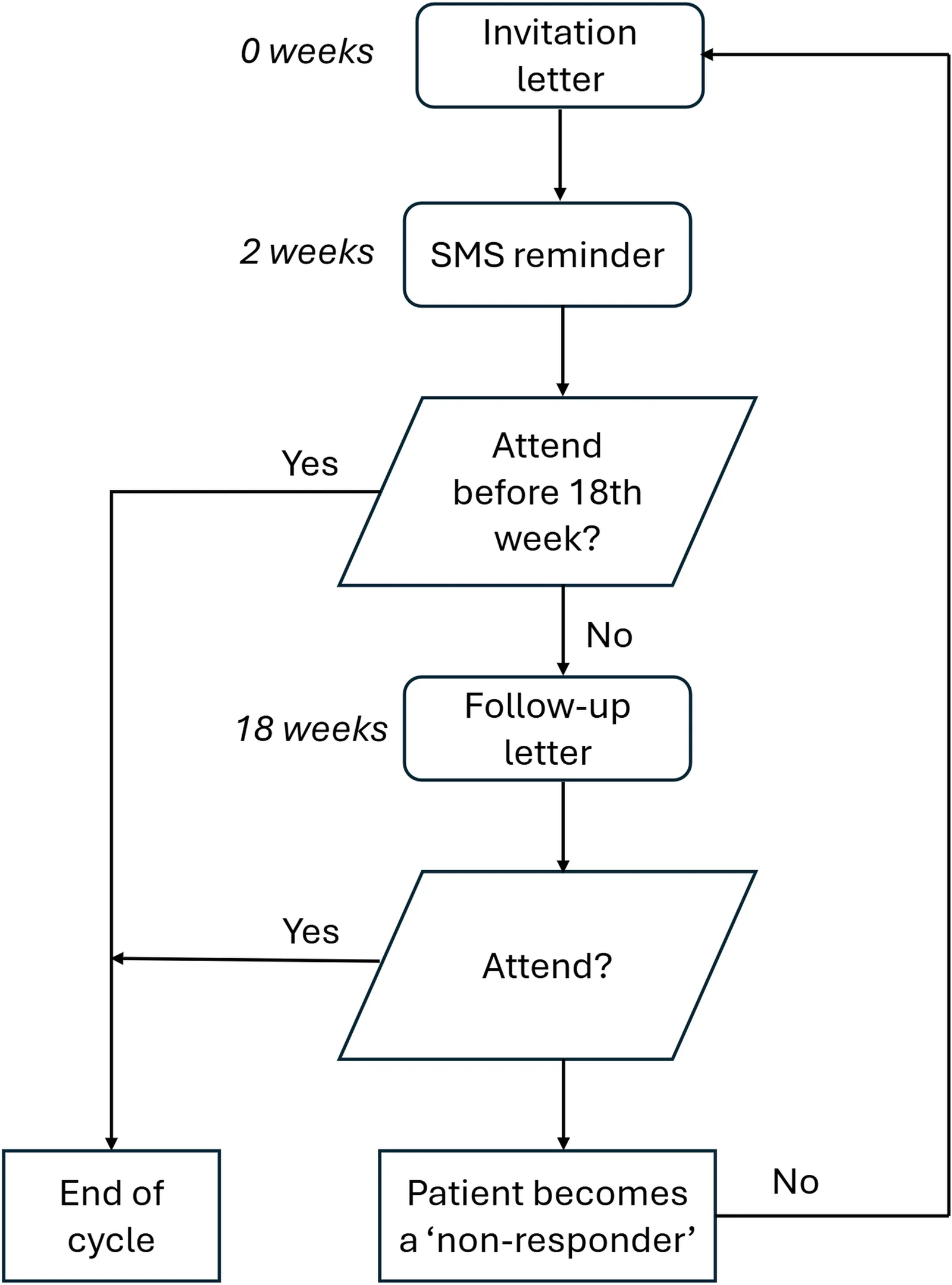
The current screening programme in England. People with a cervix recorded as female and aged between 25 and 64 are currently invited to receive cervical screening every 5 years, noting a recent change in 2025 from those aged 25–49 being invited every 3 years (which was applicable at the time of the study).

 Cervical cancer is distinctive from other cancers as it is almost entirely preventable,^1^ given that its primary cause, persistent infection with carcinogenic human papillomavirus (HPV) types, is well established and can be prevented through HPV vaccination.^2 3^ In conjunction with vaccination, cervical screening (whether testing for HPV in the first instance or not) enables the identification and treatment of precancerous lesions, interrupting progression to invasive disease and making cervical cancer largely preventable at a population level.^2 3^ Indeed, the WHO’s global strategy seeks to remove cervical cancer as a public health concern (fewer than 4 incidences per 100 000 people with a cervix).^3^ Many high-income countries are successfully pursuing the goal of tackling cervical cancer. For example, the UK’s screening (introduced from 1988, see figure 1 for the invitation process) and vaccination programmes (introduced from 2008 for girls aged 12–13 years) have significantly reduced cervical cancer incidence and almost eliminated cervical cancer in the vaccinated cohort (those born after September 1995).^4^ However, unlike breast and bowel cancer screening, uptake for cervical screening in England has decreased gradually since 2010.^5^ As of 2024, uptake rates of invited individuals aged between 25–49 and 50–64 were 66.1% and 74.3%, respectively,^5^ and uptake is not equal across the population.^6^ Cervical screening remains imperative to eliminate cervical cancer at the population level, particularly in the unvaccinated cohort.^2 3^

Addressing inequalities in cervical screening uptake is essential if the WHO’s aim of eliminating cervical cancer as a public health problem is to be met.^3^ Individuals with severe mental illness (SMI, including schizophrenia, bipolar affective disorder and other psychoses) show a higher risk of invasive cervical cancer, partially explained by a lower cervical screening participation compared with the general population.^7^ Specifically, individuals with SMI are at 11% lower odds of attending cervical screening.^8^

While many of the barriers to cervical screening attendance are similar in people living with SMI compared with the general population, including lack of knowledge about the benefits of screening, embarrassment and fear,^9 10^ the latter may be heightened in people with SMI for several reasons. In addition to well-established structural and service-based barriers,^9 11 12^ individuals with SMI often experience fear of a distressing screening experience or of receiving ‘bad news’,^12^ and are more likely to have experienced interpersonal violence^13^ and subsequently live with interpersonal trauma and trauma-related disorders,^14^ when compared with the general population.

To attempt to address these inequalities, an informed choice tool, in the form of a leaflet,^15^ was co-produced with service users with SMI, healthcare professionals and key decision-makers^16^ to address key barriers faced by people with SMI. The leaflet comprises seven sections which provide information and suggest actions the individual and their healthcare professional can take to make the process more comfortable. An accompanying animation is available that guides users through engaging with the leaflet.^17^ The preliminary evaluation of the leaflet found it to reduce decisional conflict (helped users to make a decision about whether to attend screening or not), but was underpowered to detect statistical significance in magnitude of the effect and direction of the decision.^16^ The leaflet was subsequently incorporated into UK government guidance as a webpage, with the intention to support anyone who feels anxious about attending their cervical screening.^18^ The guidance broadly follows the same structure and content as the original leaflet, but with added informative content and links to further government and National Health Service (NHS) guidance. To support implementation of the leaflet in practice, we sought to identify a feasible method of delivering the leaflet (via the government guidance webpage) to individuals with SMI, and to evaluate whether this could improve cervical screening attendance in this low-uptake population.

To reduce inequalities in screening attendance, it is imperative to ensure that the leaflet is delivered to, and benefits, people with SMI. Given that people with SMI often report negative experiences with healthcare professionals, it was important in the development phase of the leaflet that people with SMI could access it themselves, and discuss it with friends and family.^16^ Text message reminders are a relatively low-cost intervention which have already been found to increase uptake of cervical screening in the general population.^19 20^ Across two randomised controlled trials (RCTs), Huf *et al* demonstrated that General Practice (GP)-endorsed SMS message reminders (containing the name of their GP practice) significantly increased cervical screening attendance.^19^ Subsequently, GP-endorsed SMS message reminders were adopted across London and were estimated to have increased cervical screening uptake by 4.8%; an equivalent of 13 400 more women being screened.^21^ By tailoring the message to provide additional support for people with SMI, text message reminders may provide an opportunity to help to address the current disparity in cervical screening uptake. We therefore developed an enhanced GP-endorsed SMS reminder for people with SMI, which included tailored behavioural content through public and patient involvement (PPI) and a link to the leaflet in the form of the government guidance webpage.

There is a lack of RCT evidence on the use of interventions to increase cancer screening individuals SMI.^22^ To our knowledge, there is no existing robust evaluation of an intervention to increase cervical screening in individuals with SMI.^23^ Thus, this study aimed to prepare for a definitive RCT comparing the effectiveness of the standard GP-endorsed SMS message reminders with an enhanced version (containing behaviourally informed content and links to the co-produced further support) on cervical screening attendance. Specifically, this study focused on individuals with SMI who are overdue their cervical screening, as a group that is particularly disengaged with cervical screening. A pilot trial was required to establish number of eligible participants within GP practices, opt out rates, feasibility of recruiting GP practices and whether non-responders (eligible individuals who have not responded 14 weeks after receiving a reminder letter^24^) with SMI could receive and engage with texts with links to further support. Thus, the aim of our current study was to assess the feasibility of conducting an RCT that compares enhanced SMS cervical screening reminders with usual SMS reminders to increase screening uptake among individuals with SMI who are overdue their cervical screening. Specific study objectives were to assess the feasibility of recruitment, implementation of the intervention and retention of participants through GP practices (through either failure to retrieve patient details by participating GP practices, or participants opting out), as well as to rehearse data analysis effectiveness and economic evaluation to refine for a subsequent definitive trial.

## Methods

A two arm (control or intervention SMS) parallel-group pilot RCT design (with 1:1 allocation) was conducted to rehearse and test processes for a definitive trial. The trial is registered with ISRCTN (ISRCTN12558681, registered 22 October 2024). The predefined protocol for the study (see online supplemental material 1) was uploaded to Open Science Framework prior to data analysis (publicly available here: https://osf.io/gv6mr/files/nswtq). Reporting for this pilot trial followed guidance from the Consolidated Standards of Reporting Trials (CONSORT) extension for randomised pilot and feasibility trials^25^ (see online supplemental material 2 for the completed reporting checklist).

Between the conception of the study and the intervention period, the government guidance (to be included in the intervention SMS messages) was included in the standard NHS Cervical Screening Administration Service (CSAS) invitation letter SMS messages. However, due to its physical format, it was still judged to be meaningful to embed a link to the same guidance within SMS message reminders of this trial, where it could be accessed more easily. Thus, to confirm this, a survey measuring intervention fidelity was used to assess whether the government guidance was viewed and how it was accessed.

### Public and patient involvement

People with experience of mental ill health (n=7) and carers of people with SMI (n=1) were involved throughout the development of the study recruited through colleagues that had worked with individuals in this target population group before and via existing PPI networks. The group provided insights on the enhanced SMS content (advising framing around social norms) and potential barriers to opening the links (including distrust of hyperlinks), which informed feasibility outcomes relating to engagement with the links (see the description of the survey below). A PPI representative contributed to trial oversight and planned dissemination.

### GP practice recruitment

GP practices across North East and South East London were recruited between November 2024 and February 2025. Initially, all GP practices in Lambeth (South East London) were invited to be involved in the study. However, following low uptake and a low number of eligible participants from the few participating GP practices, a gatekeeper (a Clinical Lead for Population Health Management and Health Inequalities) was engaged in North East London, and purposive sampling was applied to invite GP practices with high numbers of eligible participants. Practices were approached via email (including facilitated by the gatekeeper in North East London), telephone and regional networks (eg, practice manager networks, London Cancer Alliances). In total, 13 GP practices were recruited out of 49 that were approached.

### Participant eligibility and identification

Eligible participants were people with a cervix aged 24–64 years with an SMI diagnosis who were at least 6 months overdue for cervical screening (which we define as non-responders) according to national call–recall intervals, who were registered at 1 of the 13 participating GP practices. Exclusion criteria were: ineligibility for screening (eg, pregnancy, palliative care), individuals who had recently declined screening when invited by their GP or ceased invitations via the screening service (https://csas.nhs.uk/forms/screening-cease-opt-out-gp-practice-colp/), those with type 1 SMS opt-outs, type 2 opt-outs (data-sharing dissent) and any patient judged inappropriate to be included by their GP.

A prespecified computer code containing the search terms (eligibility criteria) was developed by an experienced GP to allow GP practices to search for eligible participants via Egton Medical Information Systems (EMIS) (see table 1 the specific search terms).

**Table 1:** Summary of how the prespecified computer code operationalised the eligibility criteria

Included (all required)	Excluded (any of the below)
Screening age 6 months or more overdue their cervical screening (≥3.5 years for ages 24–49, ≥5.5 years for ages 50–64). On the Quality and Outcomes Framework SMI register.	No cervix. On the palliative care list. Ceased invitations from the CSAS and not attended screening since they joined the opt-out list. Declined screening in the last 12 months. Recorded that a smear was not indicated in the last 12 months (eg, if the patient is terminally ill or is undergoing chemotherapy, rarely recorded in around ~1–2/1000 patients). Currently pregnant. Had opted out of receiving SMS messages. Had opted out of their data being shared by their GP practice for purposes other than their own direct care (including research).

CSAS, Cervical Screening Administration Service; GP, general practice; SMI, severe mental illness.

Given that this pilot study aimed to assess feasibility rather than a definitive effect, no formal sample size was conducted. Instead, the target sample size was set based on existing guidance (a minimum of 35 participants per arm),^26 27^ with an additional 20% threshold to account for estimated opt-outs. Thus, the target was at least 88 (44 per arm), although given that the outcome was binary, the aim was to achieve 60 participants per arm (N=120).^26^

### Control

The control arm received the usual NHS SMS GP-endorsed cervical screening reminder (see below; (practice name) and (GP number) were replaced with the participants’ GP practice name and telephone number) which was implemented across London following the previous literature supporting its effectiveness.^19 20^ An opt out link for the study was added to both intervention and control texts.

(Practice name): You have not had your cervical screening test. Please call (GP number) to book. Info at https://mygp.care/CSLeaflet Privacy notice: mygp.me/CS-privacy This is part of a research study, click here for more info or to opt out: (opt-out link).

### Intervention

The intervention arm received an enhanced SMS reminder, which incorporated wording framed around social norms to signpost to the government guidance and animation via links. The remaining elements of the reminder remained the same.

(Practice name): You have not had your cervical screening test. Please call (GP number) to book. Info at https://mygp.care/CSLeaflet Worried about attending? Other people have found this animation (link to animation) and guidance (link to guidance) helpful for tips and support. Privacy notice: mygp.me/CS-privacy This is part of a research study, click here for more info or to opt out: (opt-out link).

### Study procedures

Participating practices were asked to apply prespecified computer code containing the search terms (eligibility criteria) to their list of patients to identify eligible patients for the study. GPs at the participating practices were asked to review the list of eligible patients and remove any patients they deemed inappropriate to approach at their discretion (eg, because they were experiencing psychosis). Numbers of patients excluded at this stage were recorded.

GP practices provided iPLATO with NHS numbers and dates of birth of eligible patients for randomisation and SMS delivery. Participating practices were advised to disseminate a poster informing patients about the study, and with details of opting out, which were removed before randomisation. When the target recruitment number was met, participants were randomised 1:1 to the intervention or control arm. A random participant ID (PID) for each participant was created using the excel formula ‘=RANDBETWEEN(1000000000,9999999999)’. The randomly allocated PIDs were then organised from lowest to highest, and the intervention condition was applied alternatingly (alternating between intervention and control). Both participants and GP staff were blinded to the group allocation. All messages were sent their allocated SMS on 13 March 2025 on a Thursday to optimise engagement.^20^ Participants could opt out of the study at this stage via a link in the SMS reminder.

At the end of the trial period (17 July 2025), iPLATO sent participant GP practices a list of participants from each practice, and practice staff extracted and added prespecified demographic and clinical data from GP records via EMIS (between 18 July and 2 October 2025) using another prespecified computer code, which extracted; age, Black and Minority Ethnic (BAME) ethnicity (yes/no), Index of Multiple Deprivation (postcode), date of SMI diagnosis (year), at least one long-term condition on the Quality and Outcomes Framework disease register (yes/no), and date of last cervical screening appointment (DD/MM/YYYY). GP practices pseudonymised the data before securely sending it to the researchers. After the end of the trial period, iPLATO sent all participants a brief survey on 18 July 2025 assessing whether they accessed cervical screening information (invitation letter link for control, supportive materials for intervention). The link to complete the survey was live for 30 days, and participants were sent a reminder to complete it 1 week after it was sent. At the end of the 30 days, iPLATO sent the survey responses to the researchers. In addition to the survey data, iPLATO provided feasibility metrics (eg, delivery status, invalid numbers, link clicks). Resource-use information was collected via proformas completed with both GP staff and iPLATO to inform feasibility and economic analyses.

### Outcomes

The primary outcome was feasibility, divided into four categories with constituent measures; (1) retention and recruitment rates (measured by: eligible patients per practice, patients that were excluded or opted-out, invalid or missing mobile numbers and recruitment and retention rate of GP practices), (2) data completeness (measured by: missing primary outcome data), (3) achieving comparable groups (measured by: demographic and clinical characteristics of participants for each arm and cervical screening attendance for each arm) and (4) fidelity (measured by: survey response rate, number of link clicks to the guidance and animation in the intervention arm and SMS message delivery). SMS delivery status depended on the availability of valid mobile numbers and a mobile that was turned on and receiving SMS. Number of SMS lost to scheduling was attributable to one of three reasons; (1) the patient was registered at the practice when the initial report was provided but then moved to another practice, (2) the patient changed their mobile number without notifying their practice or (3) the patients was out of the country or their phone was not connected during SMS message scheduling by iPLATO.

Secondary outcomes related to candidate measures of effectiveness for a definitive RCT and included cervical screening attendance within the 18-week trial period (13 March to 17 July). During the development of this study, it was discovered that the other candidate measure of effectiveness, stated in the protocol as the proportion of participants that had scheduled a cervical screening appointment during the trial period, was not able to be collected as details of upcoming screening appointments are not recorded on GP records. Other secondary outcome measures included resource use associated with study processes and SMS delivery. Data on whether participants accessed supportive information materials and how useful they found them were also collected to inform future implementation and adherence measures.

### Data analysis

Preparation for data analysis included converting postcodes into Index of Multiple Deprivation (IMD) decile and coding for whether cervical screening was attended within the 18-week trial period (discerned from date of last screening appointment). Analyses followed a predefined statistical analysis plan which was finalised before data analysis, publicly available on Open Science Framework (available here: https://osf.io/gv6mr/files/qutdw). As a pilot trial, primary analyses were descriptive. Baseline characteristics and feasibility measures were summarised using counts, percentages, means and SDs. Secondary data analysis rehearsed the preliminary analyses planned for a full trial, using exploratory logistic regression to assess the association between trial arms and uptake before and after adjusting for age and ethnicity. ORs and CIs were reported. iPLATO only sent participants back to their respective practice if their assigned SMS message was delivered. Thus, by nature the analyses were per-protocol, and intention-to-treat analysis was not possible. All analyses were conducted using Stata (V.19.5).^28^ Missing data were summarised with recorded reasons, but missing outcome data were not imputed.

Progression criteria were not developed a priori. Instead, the findings were interpreted within the discussion section considering benchmarks commonly used in pilot trials, focusing on criteria with existing benchmarks and thresholds and less on more subjective criteria.

## Results

### Recruitment, retention and data completeness

**Figure 2:**
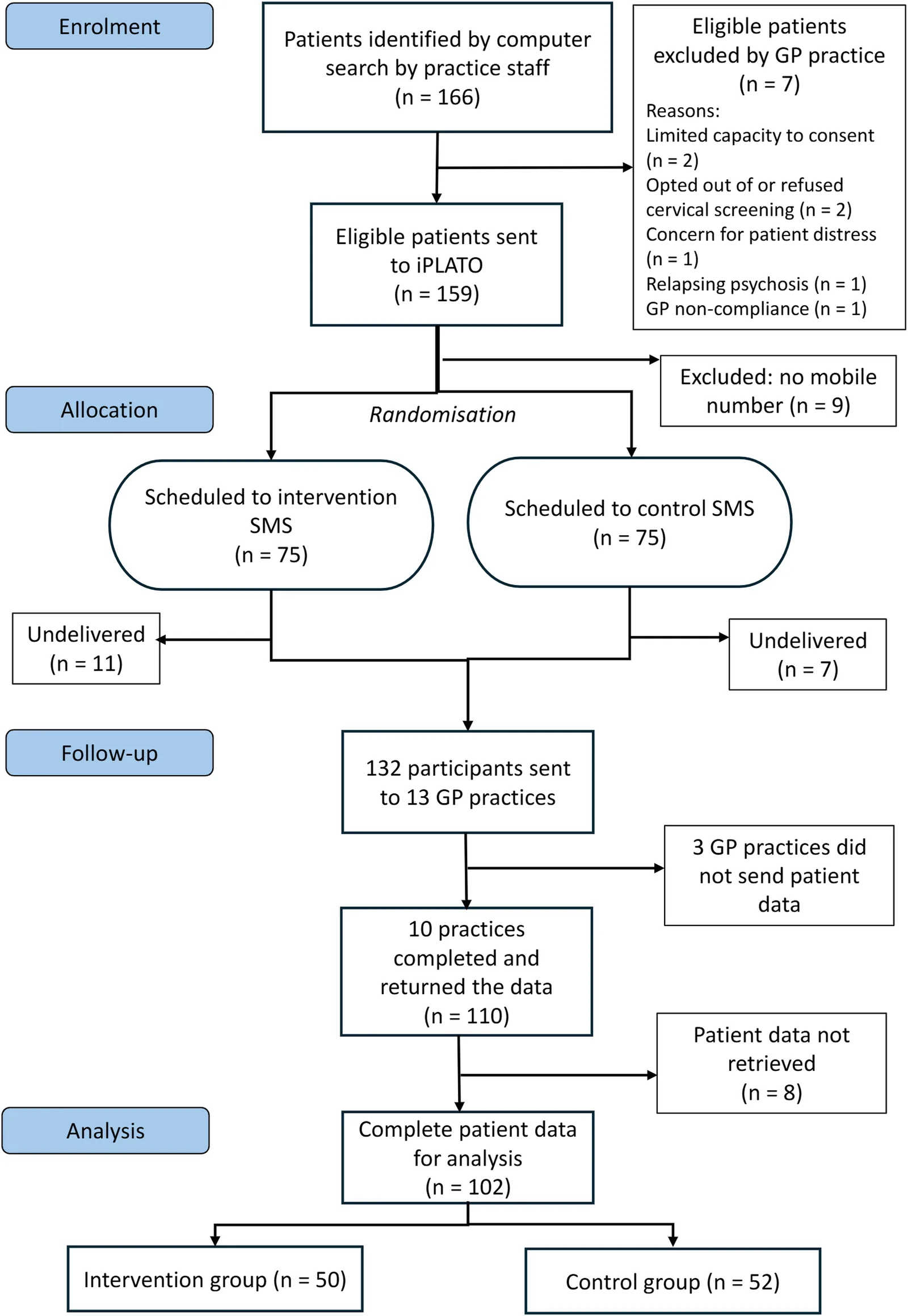
Adapted Consolidated Standards of Reporting Trials diagram of participant flow. GP, general practice; iPLATO, text messaging service company name.

14 GP practices were initially recruited across 3 months. One did not return the required information and governance and thus was excluded. The flow of participants (adhering to CONSORT^29^ guidelines for reporting) is displayed in figure 2. GP practices varied broadly in the number of eligible patients that were included in the study, ranging from 5 to 31 per practice (median=9). Seven patients were removed at the discretion of the GP practices (by only three GP practices, see figure 2 for reasons). No participants opted out. 150 participants were randomised to either the control group (n=75) or intervention group (n=75), of which 132 (88%) received an SMS. 10 (76.9%) of participating practices completed the extraction search for patients’ data and sent the complete patient datasets to the authors. Thus, outcome and demographic data were available for 64.2% of the participants who were initially randomised. The full dataset was available for 102 participants (intervention=50, control=52).

### Achieving comparable groups

Characteristics of the sample are shown in table 2. The sample characteristics for each arm were relatively similar. However, it is noteworthy that the participants in the control arm were much more likely to have one or more comorbid long-term conditions.

**Table 2:** Comparison of participant characteristics between intervention and control group. Note: data were captured after the 18-week trial period, thus participants were eligible at the time of randomisation (eg, participant/s aged 65 were 64 at the time of randomisation)

	Intervention (n=50)	Control (n=52)	Overall (n=102)
Age (years) at randomisation:			
Mean (SD)	47.5 (9.9)	46.4 (13.6)	46.9 (11.9)
Median (IQR)	45.5 (40–56)	48 (32.5–59.5)	47 (39–57)
Range: Min-Max	25–65	26–64	25–65
Ethnicity			
White	32 (64)	34 (65)	66 (65)
BAME	17 (34)	18 (35)	35 (34)
Missing status	1 (2)	0 (0)	1 (1)
Index of Multiple Deprivation (continuous: mean (SD))(range)	4.3 (2.3) (2–10)	3.9 (1.9) (2–9)	4.1 (2.2) (2–10)
10% (in top 10% deprived area)	0 (0)	0 (0)	0 (0)
20%	12 (24)	15 (29)	27 (26.5)
30%	12 (24)	10 (19)	22 (21.5)
40% or less in deprived area	26 (52)	25 (52)	53 (52)
Years since SMI diagnosis (n=97)	(n=47)	(n=50)	(n=97)
Mean (SD)	12.6 (8.2)	12.5 (9.6)	12.5 (8.9)
Median (IQR)	12 (7–16)	9.5 (5-18)	10 (6–16)
Range: Min-Max	1–47	1–48	1–48
At least one long-term condition on the QoF disease register			
Yes (%)	11 (22)	25 (48)	36 (35)
No (%)	39 (78)	27 (52)	66 (65)
Screening status during intervention period (Y/N)			
Yes (%)	4 (8)	1 (2)	5 (5)
No (%)	46 (92)	52 (98)	97 (95)

BAME, Black and Minority Ethnic; QoF, Quality and Outcomes Framework; SMI, severe mental illness.

### Fidelity

**Figure 3:**
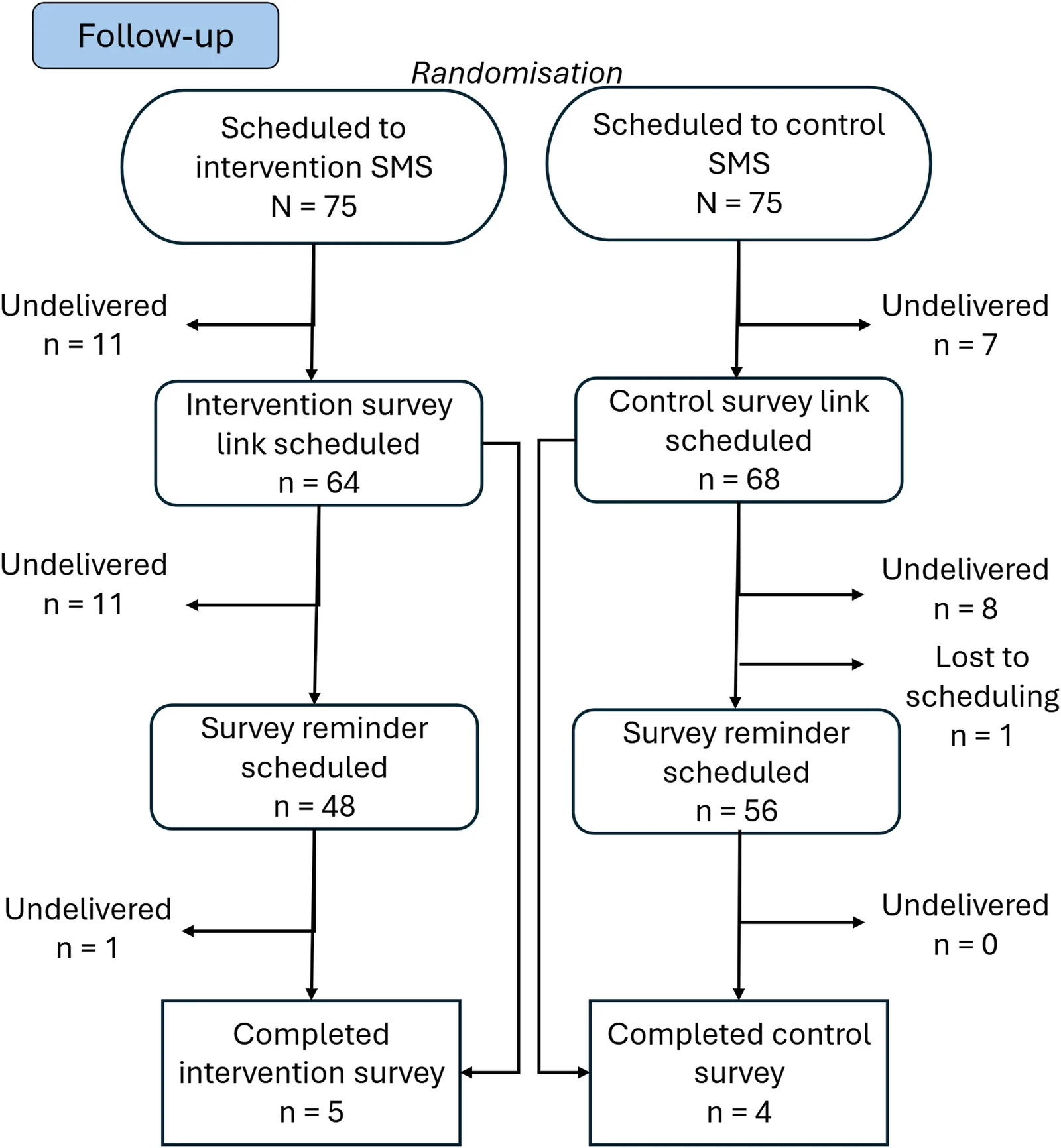
Adapted Consolidated Standards of Reporting Trials diagram of the delivery and response rate of the survey.

Of the SMS messages sent, most were delivered (64/75 91% in the intervention and 68/75 85% in the control arm). As shown in figure 3, only 8% of those who received the survey link responded (5% in the control group n=4, and 9% in the intervention group n=5).

The animation and guidance included in the intervention arm received 37 and 5 unique clicks, respectively. Comparatively, the links in both SMS messages (the general government cervical screening information and the privacy notice) received 42 and 13 unique clicks, respectively. The utility of the link click data was limited, as clicks were not separated by arm and included multiple clicks from the same participant. The nine responses to the survey indicated limited engagement with the resources. Most control arm participants did not remember receiving the cervical screening invitation letter (n=3/4), while the remaining participant chose not to access the guidance link because they did not wish to attend cervical screening. By contrast, most participants in the intervention arm recalled receiving an invitation letter (4/5), although only one participant remembered accessing the link within the letter. Similarly, most intervention arm participants (4/5) did not access (n=3) or could not remember accessing (n=1) the guidance and animation links sent via SMS. The single participant who accessed these resources rated them as very helpful, on a 5-point Likert scale from ‘not at all helpful’ to ‘extremely helpful’. One participant did not access the further support because they experienced a relapse of their mental health condition. The only free text comment enquired about home visits for screening.

### Rehearsal of analysis of outcomes

The odds of attending for a cervical smear were on average higher in the intervention arm than in the control (OR=4.43, 95% CI 0.48 to 41.13); however, the CI was very wide and consistent with both an increase or decrease in attendance. The extent of the uncertainty was influenced by the very low uptake of screening; just 5 out of the 102 in the trial (4 in intervention and 1 in the control). As would be expected, a similar lack of evidence was found when the OR estimate comparing arms was additionally adjusted for age and ethnicity (OR=4.60, 95% CI 0.49 to 43.03). Findings of the data collection required for an economic evaluation in a definitive trial are reported in online supplemental material 3. In summary, the intervention text cost 50% more than control texts (5p vs 7.5p per SMS). Out of all required tasks, GP practices spent the most time extracting patient data (an average of 158 min).

## Discussion

In the absence of prespecified progression criteria, the study’s feasibility is interpreted by referencing progression criteria from other external pilot and feasibility studies;^30 31^ thus, post hoc interpretation of feasibility should be cautious. Overall, feasibility was mixed: targeted delivery of enhanced SMS message reminders to underserved or under-engaged patient groups is feasible, and practice retention was acceptable. However, participant retention through GP practices and data completeness fell below commonly used progression criteria, indicating that feasibility is limited without addressing participant retention through GP practices. Alternative methods (to surveys delivered by SMS) are required to understand whether and how patients might engage with resources, including outside of the intervention SMS message (and the invitation letter). Additionally, the persistence of low service uptake outcomes, while to be expected in a population recruited based on prior non-engagement, means that any subsequent trial would need to be substantial in size to detect effects on outcomes. Lastly, the persisting low cervical screening attendance indicates that even if evidenced to be effective in future trials, enhanced messaging would likely be only part of a multicomponent approach to supporting cervical screening uptake for non-responders living with SMI. Concerns about interoperability of research data collected from different GP practices were also evident, raising questions about the feasibility of these data collection methods for any future trial. Some feasibility outcomes are discussed in further detail below (see table 3). In sum, with an alternative approach to data collection of outcome and fidelity data, stratified randomisation and continued use of a gatekeeper-led recruitment approach, progression to a definitive trial may be feasible.

**Table 3:** Feasibility outcomes and recommendations for a definitive RCT. Existing progression criteria are only referenced where comparisons a posteriori are possible and useful

Outcome	Measure	Finding	Feasibility (referenced criteria, if applicable)	Resulting recommendation for a definitive trial
Recruitment and retention	GP practice recruitment rate	27% of GP practices that were individually approached were recruited.	GP practice recruitment was feasible with assistance from a gatekeeper.	Use a gatekeeper to identify GP practices with high numbers of eligible participants and facilitate recruitment of GP practices.
	GP practice retention rate	76.9% of recruited GP practices were retained throughout the study.	Satisfactory, compared with a goal of 88% or higher when compared with progression criteria outlined by a pilot RCT that also included recruitment of GP practices.^30^	Minimise workload of GP practices by exploring other methods of data entry (see below).
	Participant retention through GP practices	No participant opt-outs. Participants were instead lost through GP practices not retrieving patient data or patients’ data being unable to be retrieved.	Opt-out model of participant recruitment is feasible.	Amend process of adding patient data (eg, ensure interoperability of search terms across GP systems or centralise retrieval).
Achieving comparable groups	Randomisation into comparable groups	More participants in the control arm had one or more comorbid long-term conditions.	Should be explored for a definitive trial	Stratify (eg, by presence of comorbidities) to remove demographic confounders.
	Cervical screening attendance	Low uptake of cervical screening attendance across both groups (8% of the intervention group and 2% of the control), resulting in large CIs.	Would likely require a large sample size to detect a significant difference within a fully powered trial.	Consider broadening the study sample to individuals with SMI or non-responders in general to decrease the required sample size of a definitive trial.
Fidelity	Survey completion rate	Survey completion rates were extremely low (5% in the control group and 9% in the intervention group).	Use of a survey delivered via SMS message was not feasible to assess fidelity.	Adopt other ways to assess intervention fidelity (while still minimising interference with participants), eg, (1) improve link click detection and analytics data from the links. (2) Collect further data from participating GP practices about impact of the study (any changes in screening consultations with the target group ask them to note down if anyone asked about additional requirements, etc before/after.
	SMS delivery	SMS messages delivered to 88.0% of eligible participants.	Higher than expected compared with delivery rates within RCTs of SMS message reminders delivered to the general population,^19^ which ranged from 31.5% to 41.2% of SMS messages delivered.	N/A
Data completeness	Completeness of effectiveness outcomes	Patient data completeness was 64.2%.	Unsatisfactory, when compared with both when compared with progression criteria outlined by a pilot RCT (a goal of 80% or higher) that also included recruitment of GP practices,^30^ and a range of internal pilot studies which ranged from a minimum of 75–85% participants with follow-up outcome data.^31^	Collect date of last cervical screening appointment before and after the trial period.
	Completeness of cost-effectiveness outcomes	60% (6/10) GP practices completed cost data.	Likely should be improved for a definitive trial, to ensure representativeness.	Encourage participating GP practices to fill out the primary care resources tool, so that job roles are correctly allocated to their corresponding cost per task.

GP, general practice; N/A, not applicable; RCT, randomised controlled trial; SMI, severe mental illness.

Delivery of SMS messages was high relative to two previous RCTs testing the effectiveness of SMS message reminders in the general population reporting only 31.5–41.2% of SMS messages delivered, depending on the age group.^19^ Though any explanation would be speculative, the high delivery rates are possibly explicable due to individuals with SMS living with frequent health needs and are thus in regular contact with GP practices, including for annual health checks.^11^

The main barrier to participant retention through GP practices identified in the current study was that GP practices encountered barriers in extracting and adding patient data due to technical difficulties attributable to a lack of interoperability across GP practice systems, which created a challenge around developing a computer code that applied to all systems. Interoperability of healthcare technology systems is not only a barrier to research, but also delivery of health and social care services.^32^ Consistency, or at least compatibility, of GP information technology systems across GP practices is essential for integration, for both research and practice.

This study explored just one approach to delivering the informed-choice leaflet to people with SMI. The high delivery rate of the SMS reminders indicates it to be effective at reaching people with SMI, although engagement with the links was unclear. The low observed attendance during the trial period is perhaps expected, given that participants were both living with SMI and classed as non-responders. Also, given that the response to the survey was so low, it is unknown whether participant engaged with the links to the further support. Further exploration is needed to explore if the enhanced SMS messages help people with SMI to make informed decisions, as intended.^16^ Also, the leaflet likely addresses only some of the most common barriers to cervical screening, specifically barriers around fear and embarrassment related to cervical screening, for individuals already considering whether to attend screening.^16^ However, further and more intensive intervention is still needed for individuals living with the trauma of previous abuse and people living with psychoses, who are unlikely to consider screening at all.^16 33^ At-home self-sampling cervical screening could help to address other remaining practical barriers such as childcare issues,^33^ enabling individuals who do not have the capacity to attend an appointment to have access to cervical screening. Future research is needed to explore the acceptability and accessibility of at-home self-sampling for people with SMI.

Given the small additional cost of the enhanced SMS message and that the leaflet helps address common barriers to cervical screening in the general population including feeling anxious about attending due to fear and embarrassment and low health literacy around the purpose of cervical screening,^10^ it is possible that the enhanced text message could replace the current standard SMS message reminders. Indeed, this approach may prove to be lower cost than searching for individuals with SMI who are overdue. However, the merits of a population-wide approach must be considered with regards to proportionate universalism, where support should be scaled up proportionate to level of advantage to reduce health inequalities.^34^ Another approach is to send the enhanced SMS messages to all non-responders, similar to the approach taken in a previous evaluation, which provided non-responders with an educational video via SMS and email, as well as an online booking system via SMS.^33^ As such, cost-effectiveness must be central to future evaluations to inform whether a specific or population-wide approach is most appropriate.

It is important to note that the current study included the government guidance, adapted from the original informed choice leaflet, within the intervention SMS message reminders. However, there are elements and behavioural content that are missing within the government guidance, which may be key to the mechanisms of reducing decisional conflict. For example, in the conceptualisation and proof-of-concept work for the leaflet, the checklist, where individuals can select the things that they struggle with about attending cervical screening which can be shared with their healthcare practitioner was identified to be particularly important.^16^ This checklist is merely signposted to as a link on the online government guidance and requires printing, potentially limiting the impact and utility of the leaflet. Also, the original leaflet included quotes from peers, which may be an important element given that social norms were identified as important in the Patient and Public Involvement and Engagement (PPIE) work during the current study. To be inclusive to anyone who feels anxious about attending, specific references to SMI were excluded (eg, the ‘reasons for extra support’ section initially included I hear voices), which may have helped people with SMI to feel recognised and adequately supported. The original leaflet also went through extensive testing for readability, assessed using the Flesch Reading Ease and Flesch-Kincaid Scales.^35 36^ The government version is written more formally and is less readable. Combined with being displayed on a government website, this may mean that the government guidance was less relational, particularly as individuals with SMI often report lack of trust in public sector services.^37^

### Strengths and limitations

This pilot study examined the feasibility of conducting the first evaluation of an intervention to increase cervical screening uptake in people with SMI, contributing to the literature that aims to reduce the established inequality in uptake. However, several challenges were identified mostly around accuracy and quality of the extracted data from GP records, which would be important to rectify for a fully powered trial. First, the current study was unable to report on date of last cervical screening before the trial period, as the prespecified search terms which were applied after the trial period only extracted date of last screening, meaning that the data were unavailable for anyone who attended screening within the trial period. It is crucial that both dates are collected within a fully powered trial (both date of last screening before the trial period, and date of cervical screening attendance within the trial period), particularly since previous cervical screening attendance is a strong predictor of future attendance.^20^ Also, due to technical difficulties with the prespecified computer code to add patient data from EMIS, many GP practices manually extracted data from the search into the dataset, which may have introduced human error during data entry. Due to the nature of the data flow through iPLATO, an intention-to-treat analysis was not possible. Thus, for a fully powered definitive trial, it is important for the involved SMS message provider to retain participants for whom the SMS message does not deliver for, so they can be included in an intention-to-treat analysis. Finally, date of SMI diagnosis only provides a proxy, due to (1) only the year being extracted to protect the anonymity of participants and (2) poor integration and communication between primary and secondary care. Additionally, the lack of prespecified progression criteria hindered objective assessment of feasibility of a definitive RCT. Finally, the relatively low GP practice recruitment rate (27%) may indicate possible selection bias for a definitive trial. However, the systematic selection of participants within each GP practice using the opt-out model arguably minimises the opportunity for bias, given that selection bias in GP practice research is mainly present in the characteristics of participating GP practices (compared with non-participating practices) and in the patients that GPs recruit, rather than any differences between patients registered at participating and non-participating practices.^38 39^ A more noteworthy limitation is that this study was conducted in London, which may differ to the rest of the UK in terms of GP practice engagement in research,^40^ affecting GP recruitment in a larger definitive trial.

## Conclusion

Behaviourally informed SMS reminders can be delivered to provide further support to people with SMI, although it is uncertain if the extra resources are accessed and used. A full trial is not yet feasible without major methodological adjustments, particularly around data extraction and participant follow-up. The complexities of searching heterogeneous GP computer systems should be addressed for more efficient research and practice. Future research is needed to explore other interventions (including more intensive interventions) that can address other barriers to cervical screening in individuals in SMI, and how these may complement the current intervention approach to optimise impact.

## Supplementary material

10.1136/bmjopen-2026-122934 online supplemental file 1

10.1136/bmjopen-2026-122934 online supplemental file 2

10.1136/bmjopen-2026-122934 online supplemental file 3

## Data Availability

No data are available.

## References

[1] Wu J, Jin Q, Zhang Y (2025). Global burden of cervical cancer: current estimates, temporal trend and future projections based on the GLOBOCAN 2022. *J Natl Cancer Cent*.

[2] Wentzensen N, Schiffman M (2018). Accelerating cervical cancer control and prevention. Lancet Public Health.

[3] World Health Organization (2020). Global strategy to accelerate the elimination of cervical cancer as a public health problem.

[4] Falcaro M, Castañon A, Ndlela B (2021). The effects of the national HPV vaccination programme in England, UK, on cervical cancer and grade 3 cervical intraepithelial neoplasia incidence: a register-based observational study. The Lancet.

[5] Office for Health Improvement and Disparities (2025). Fingertips: cancer screening. https://fingertips.phe.org.uk/search/cancer%20screening.

[6] Abraham S, Foreman N, Sidat Z (2022). Inequalities in cancer screening, prevention and service engagement between UK ethnic minority groups. Br J Nurs.

[7] Hu K, Wang J, Sparén P (2023). Invasive cervical cancer, precancerous lesions, and cervical screening participation among women with mental illness in Sweden: a population-based observational study. Lancet Public Health.

[8] Solmi M, Firth J, Miola A (2020). Disparities in cancer screening in people with mental illness across the world versus the general population: prevalence and comparative meta-analysis including 4 717 839 people. Lancet Psychiatry.

[9] Aggarwal A, Pandurangi A, Smith W (2013). Disparities in breast and cervical cancer screening in women with mental illness: a systematic literature review. Am J Prev Med.

[10] Chorley AJ, Marlow LAV, Forster AS (2017). Experiences of cervical screening and barriers to participation in the context of an organised programme: a systematic review and thematic synthesis. *Psychooncology*.

[11] Owens J, Ravindrarajah R, Norman G (2025). Access, inequalities and annual health checks (AHCs) for adults living with severe mental illness in the UK: a mixed-methods systematic review. BMJ Open.

[12] Tuschick E, Barker J, Giles EL (2024). Barriers and facilitators for people with severe mental illness accessing cancer screening: A systematic review. *Psychooncology*.

[13] Khalifeh H, Oram S, Osborn D (2016). Recent physical and sexual violence against adults with severe mental illness: a systematic review and meta-analysis. *Int Rev Psychiatry*.

[14] Mauritz MW, Goossens PJJ, Draijer N (2013). Prevalence of interpersonal trauma exposure and trauma-related disorders in severe mental illness. Eur J Psychotraumatol.

[15] Lamontagne-Godwin F, Barley E (2019). Support available for your cervical screening (smear test).

[16] Lamontagne-Godwin FR, Henderson C, Lafarge C (2025). Development and preliminary evaluation in community mental health teams of a cervical screening informed-choice tool for women with severe mental illness in England: a mixed-method study. BMJ Open.

[17] Animated S (2019). Support for your cervical screening (smear test). https://www.youtube.com/watch?v=75MUQVwv908.

[18] NHS England Cervical screening: support for people who feel anxious about attending 2025. https://www.gov.uk/government/publications/cervical-screening-support-for-people-who-find-it-hard-to-attend/cervical-screening-support-for-people-who-feel-anxious-about-attending.

[19] Huf S, Kerrison RS, King D (2020). Behavioral economics informed message content in text message reminders to improve cervical screening participation: Two pragmatic randomized controlled trials. Prev Med.

[20] Nichol B, Ruwende J, Powell Davies J Evaluation of the impact of London-wide behaviourally informed text message reminders on cervical screening uptake: a naturalistic study. under review.

[21] Ruwende J (2019). GP-endorsed text reminders help increase cervical screening attendance in London. https://phescreening.blog.gov.uk/2019/07/15/gp-endorsed-text-reminders-help-increase-cervical-screening-attendance-in-london/#:~:text=The%206%2Dmonth%20project%20in,the%20implementation%20of%20the%20project.

[22] Barley E, Borschmann RD, Walters P (2013). Interventions to encourage uptake of cancer screening for people with severe mental illness. Cochrane Database Syst Rev.

[23] Lamontagne-Godwin F, Burgess C, Clement S (2018). Interventions to increase access to or uptake of physical health screening in people with severe mental illness: a realist review. BMJ Open.

[24] NHS Cervical Screening Administration Service Invitations, reminders and results communications. https://csas.nhs.uk/invitations-reminders-and-results-letters/.

[25] Eldridge SM, Chan CL, Campbell MJ (2016). CONSORT 2010 statement: extension to randomised pilot and feasibility trials. BMJ.

[26] Teare MD, Dimairo M, Shephard N (2014). Sample size requirements to estimate key design parameters from external pilot randomised controlled trials: a simulation study. Trials.

[27] Whitehead AL, Julious SA, Cooper CL (2016). Estimating the sample size for a pilot randomised trial to minimise the overall trial sample size for the external pilot and main trial for a continuous outcome variable. Stat Methods Med Res.

[28] (2025).

[29] Hopewell S, Chan A-W, Collins GS (2025). CONSORT 2025 statement: updated guideline for reporting randomised trials. The Lancet.

[30] Hynes L, Murphy AW, Hart N (2022). The MultimorbiditY COllaborative Medication Review And DEcision Making (MyComrade) study: a protocol for a cross-border pilot cluster randomised controlled trial. Pilot Feasibility Stud.

[31] Avery KNL, Williamson PR, Gamble C (2017). Informing efficient randomised controlled trials: exploration of challenges in developing progression criteria for internal pilot studies. BMJ Open.

[32] McGowan LJ, Graham F, Lecouturier J (2024). The Views and Experiences of Integrated Care System Commissioners About the Adoption and Implementation of Virtual Wards in England: Qualitative Exploration Study. J Med Internet Res.

[33] Haith L, Deaney C, Reesby D (2025). A Retrospective Observational Study on the Impact of Digital Strategies to Boost Cervical Screening Uptake in Primary Care. Cancer Control.

[34] Marmot M, Bell R (2012). Fair society, healthy lives. Public Health.

[35] FLESCH R (1948). A new readability yardstick. J Appl Psychol.

[36] Kincaid JP, Fishburne Jr RP, Rogers RL (1975). Derivation of new readability formulas (automated readability index, fog count and flesch reading ease formula) for navy enlisted personnel.

[37] Smith TE, Easter A, Pollock M (2013). Disengagement from care: perspectives of individuals with serious mental illness and of service providers. *Psychiatr Serv*.

[38] Ertmann RK, Nicolaisdottir DR, Kragstrup J (2020). Selection bias in general practice research: analysis in a cohort of pregnant Danish women. Scand J Prim Health Care.

[39] Tranberg K, Due TD, Rozing M (2023). Challenges in reaching patients with severe mental illness for trials in general practice-a convergent mixed methods study based on the SOFIA pilot trial. Pilot Feasibility Stud.

[40] National Institute for Health and Care Research (2025). RDN portfolio data. https://www.nihr.ac.uk/about-us/who-we-are/reports-and-performance/annual-statistics.

