# Supplementary material for: Behaviourally informed text message reminders to increase cervical screening attendance in people with severe mental illness: the OPTIMISE pilot randomised controlled trial
Source: BMJ Open. 2026 Sep 11;16(9):e122934. doi: 10.1136/bmjopen-2026-122934 (PMC13583816; doi:10.1136/bmjopen-2026-122934)
Supplement: Supplementary file 1 [file bmjopen-2026-122934.reviewer_comments.pdf]

PEER REVIEW HISTORY

BMJ Open publishes all reviews undertaken for accepted manuscripts. Reviewers are asked to complete a checklist review form and are provided with free text boxes to elaborate on their assessment. These free text comments are reproduced below.

ARTICLE DETAILS

Title (Provisional)

Behaviourally informed text message reminders to increase cervical screening attendance in people with severe mental illness: the OPTIMISE pilot randomised controlled trial

Authors

Nichol, Beth; Bryant, Andrew; Hall, Lesley; von Wagner, Christian; Barley, Elizabeth; Grimani, Aikaterini; Oliver, Emily; Osborn, David; Vale, Luke; Vlaev, Ivo; Graham, Fiona

---

VERSION 1 - REVIEW

**Reviewer**	**1**
**Name**	**Alimoradi, Zainab**
**Affiliation**	**Qazvin University of Medical Sciences, Midwifery**
**Date**	**06-Jun-2026**
**COI**	**None**

This is a well-conducted pilot RCT addressing an important inequality in cervical screening. The topic is highly relevant, and the study design is generally sound. However, several reporting, methodological clarity, and writing issues must be addressed to comply with CONSORT extension for pilot trials and BMJ Open standards.

Mandatory Revisions

Abstract

Add primary feasibility outcomes numerically – Report key metrics: recruitment rate (27%), SMS delivery (88%), practice retention (77%), data completeness (64%), survey response (7%).

Clarify sample size – State target was 120 participants (60 per arm) based on Teare et al. (2014) guidance.

Add trial registration date – Include date of registration for ISRCTN12558681.

Methods

Prespecified progression criteria – State clearly that none were defined a priori (currently only in discussion; move to methods).

Intention-to-treat limitation – State in methods that per-protocol analysis only was possible due to iPLATO system limitations.

Define "non-responder" earlier – Move definition from page 7 to earlier in methods section.

Results

Fix inconsistent numbers – Check consistency between abstract, text, and Figure 2 for participant flow.

Table 2 – missing column header – Last column requires header: "Comorbidity (≥1 LTC)".

Add denominator for link clicks – Report clicks as percentage of delivered messages (e.g., 37/64 = 58%).

Survey response data – Move key findings from Supplementary Material 3 into main results (brief summary).

Discussion

Avoid over-interpretation of OR – Add: "The wide confidence interval (0.48 to 41.13) means no conclusion about effectiveness can be drawn."

Add clear feasibility conclusion – Add: "Based on observed data completeness (64%) and survey response (7%), a definitive trial is not feasible without major methodological changes."

Strengths and Limitations

Add limitation: no progression criteria – Currently mentioned in discussion; add to this section.

Add limitation: per-protocol analysis only – State explicitly.

Strengthen fidelity limitation – "Low survey response (7%) severely limits assessment of intervention fidelity."

Writing & Clarity Revisions

Shorten introduction first paragraph (page 5, lines 30-50) – Split into two paragraphs.

Figure 1 – Not visible in current PDF. Resubmit high-resolution image.

Figure 2 – Verify visibility; ensure CONSORT flow diagram is clear.

Table 1 – Reformat as standard grid; current formatting is broken.

Control SMS text (page 9) – Add note that "[Practice Name]" and "[GP number]" were replaced with actual details.

Typo page 11 – "decrmed" should be "derived".

Typo page 14 – "post- post-progression criteria" remove extra "post-".

Table 3 – cut-off text – Complete sentence in "Assessing fidelity" row: "...e.g., improved link click detection and analytics data."

Reduce repetition of "feasibility" – Vary with "practicability," "acceptability of methods," "operational viability."

Methodological Gaps

No prespecified progression criteria – State absence clearly and justify post-hoc interpretation.

Per-protocol analysis only – Major limitation. Recommend authors work with SMS provider to retain undelivered participants for ITT in future trials.

Low data completeness (64%) – Falls below typical 80% threshold. Discuss acceptability for SMI population.

Low survey response (7%) – Recommend alternative fidelity methods (link click analytics, GP record review).

Baseline imbalance in comorbidities – Control arm had more participants with ≥1 LTC (48% vs 22%). Recommend stratified randomisation in definitive trial (noted in Table 3 – good).

GP practice recruitment rate (27%) – Discuss potential selection bias.

No power calculation for pilot – Acceptable but state target based on published guidance.

Single geographic area (London only) – Limits generalisability.

Opt-out model – Acknowledge that participant characteristics remain unknown.

Date of SMI diagnosis as year only – Proxy measure only.

**Reviewer**	**2**
**Name**	**Garrett, Nick**
**Affiliation**	**AUT University, Department of Biostatistics and Epidemiology**
**Date**	**03-Jul-2026**
**COI**	**None**

The article is overall well written but there are some areas that need improvement.

Introduction:

The introduction lacks some detail.There is a statement that cervical cancer is almost entirely preventable, although a reference is given more detail about why it is preventable is ideally required. There is reference to screening and vaccination but it is not explicitly stated. Also

the reference to vaccination needs more detail as it is not a direct vaccination for cervical cancer.

There is a considerable part of the introduction that relates to the development of a leaflet/tool (note that there is some confusion as to whether it is a leaflet or a tool or both), however it is not fully clear whether this tool was directly used to inform the SMS intervention (or the SMS intervention content). There is also reference made to the fact that the tool appears to have been widely disseminated before the trial and therefore potentially impacting on the trial.

Methods:

Generally the methods are well laid out and detailed.However the primary outcomes as stated in the methods are not fully corresponding to what is stated in the abstract "feasibility outcomes" or results section. Namely giving detailed measures but not explicitly stating key feasibility measures such as recruitment rates, retention rates, acceptability etc.

VERSION 1 - AUTHOR RESPONSE

**Reviewer 1**	
Comments to the Author:<br>This is a well-conducted pilot RCT addressing an important inequality in cervical screening. The topic is highly relevant, and the study design is generally sound. However, several reporting, methodological clarity, and writing issues must be addressed to comply with CONSORT extension for pilot trials and BMJ Open standards.	Thank you for your detailed review and analytical feedback. We appreciate your clear directions for improved reporting, and the manuscript has significantly increased as a result.
Mandatory Revisions<br>Abstract<br>Add primary feasibility outcomes numerically – Report key metrics: recruitment rate (27%), SMS delivery (88%), practice retention (77%), data completeness (64%), survey response (7%).	Thank you for this helpful suggestion. Details of the primary feasibility outcomes has been added in the abstract as suggested:<br><br>‘*Primary feasibility outcomes of recruitment rate of GP practices (27%), SMS delivery (88%), retention of GP practices (77%), and data completeness (64%) indicated feasibility with some amendments, although survey response rate (7%) indicated that use of a survey delivered via SMS message was not feasible to assess fidelity.*’
Clarify sample size – State target was 120 participants (60 per arm) based on Teare et al. (2014) guidance.	This has been added to the abstract.
Add trial registration date – Include date of registration for ISRCTN12558681.	The date has been added in the abstract and main text.
Methods<br>Prespecified progression criteria – State clearly	This has already been noted in the data analysis section of the methods. Given that progression criteria are applied to interpret the findings, we

that none were defined a priori (currently only in discussion; move to methods).	maintain that it best fits here. The lack of progression criteria has also been added to the ‘strengths and limitations’ section after the abstract.
Intention-to-treat limitation – State in methods that per-protocol analysis only was possible due to iPLATO system limitations.	Again, this is already noted in the data analysis section of the methods. This limitation has also been added to the ‘strengths and limitations’ section after the abstract.
Define "non-responder" earlier – Move definition from page 7 to earlier in methods section.	Non-responders are defined within the last paragraph of the introduction, then again within the ‘participant eligibility and identification’ section of the methods, which we uphold is sufficient and appropriately placed.
Results<br>Fix inconsistent numbers – Check consistency between abstract, text, and Figure 2 for participant flow.	Thank you. I have double checked and the information across the abstract, Figure 2, and participant flow is all correct.
Table 2 – missing column header – Last column requires header: "Comorbidity (≥1 LTC)"	I am not sure what you mean here. The header is there for comorbidities but the section spreads across two pages. No change has been made.
Add denominator for link clicks – Report clicks as percentage of delivered messages (e.g., 37/64 = 58%).	Unfortunately, the link click data was of limited utility, and its specificity is something we would be looking to improve for the definitive trial (see the recommendation in Table 3). We have added explanation of why a denominator is not available in the results section, where the link click data is reported:<br><br>‘*The utility of the link click data was limited, as clicks were not separated by arm, and included multiple clicks from the same participant*.’
Survey response data – Move key findings from Supplementary Material 3 into main results (brief summary).	The survey findings have now been moved from the supplementary material and into the results section (under ‘feasibility outcomes’).
Discussion<br>Avoid over-interpretation of OR – Add: "The wide confidence interval (0.48 to 41.13) means no conclusion about effectiveness can be drawn."	We deliberately avoided any interpretation of the odds ratio, as examining effectiveness was not the aim of this pilot trial. I would therefore like to avoid any discussion of the odds ratio and confidence interval at all in the discussion. No changes have been made.
Add clear feasibility conclusion – Add: "Based on observed data completeness (64%) and survey response (7%), a definitive trial is not feasible without major methodological changes."	This is already present within the conclusion section, see below:<br><br>‘A full trial is not yet feasible without major methodological adjustments, particularly around data extraction and participant follow-up.’

Strengths and Limitations<br>Add limitation: no progression criteria – Currently mentioned in discussion; add to this section.	The lack of progression criteria has been added to the 'strengths and limitations' section after the abstract.
Add limitation: per-protocol analysis only – State explicitly.	The default per protocol analysis has been added to the 'strengths and limitations' section after the abstract.
Strengthen fidelity limitation – "Low survey response (7%) severely limits assessment of intervention fidelity."	This is already included in Table 3 (see the 'Assessing fidelity' row). This is not an appropriate limitation as such, since one of the aims of this pilot trial was to test whether the survey was a feasible method to examine fidelity. We have fulfilled this aim by concluding that it is not. No changes have been made.
Writing & Clarity Revisions<br>Shorten introduction first paragraph (page 5, lines 30-50) – Split into two paragraphs.	Thanks- done.
Figure 1 – Not visible in current PDF. Resubmit high-resolution image.	In line with journal guidance, Figures were submitted as a separate file (as an editable PowerPoint file). For the purpose of peer review, Figures have also been added as PDFs.
Figure 2 – Verify visibility; ensure CONSORT flow diagram is clear.	See above.
Table 1 – Reformat as standard grid; current formatting is broken.	Please note that the formatting in Table 1 is not broken. The bullet points are not intended to correspond across the included and excluded columns. Thus, no changes have been made.
Control SMS text (page 9) – Add note that "[Practice Name]" and "[GP number]" were replaced with actual details.	A note has been added:<br><br>*'(see below; [practice name] and [GP number] were replaced with the participants' GP practice name and telephone number)'*
Typo page 11 – "decrmed" should be "derived".	I am unable to find this typo.
Typo page 14 – "post- post-progression criteria" remove extra "post-".	Thank you- corrected.
Table 3 – cut-off text – Complete sentence in "Assessing fidelity" row: "...e.g., improved link click detection and analytics data."	Formatting has been amended to prevent this.
Reduce repetition of "feasibility" – Vary with "practicability," "acceptability of methods," "operational viability."	'Feasibility' is used in the text only 24 times, as a recognised term by the MRC framework. I have checked the text throughout and concluded that this is not necessary.
Methodological Gaps<br>No prespecified progression criteria – State absence clearly and justify post-hoc interpretation.	See my response to other comments about this above.
Per-protocol analysis only – Major limitation. Recommend authors work with SMS provider to retain undelivered participants for ITT in future trials.	Thank you for this suggestion- we agree for a future trial. This has already been suggested in the strengths and limitations section:

	'for a fully powered definitive trial, it is important for the involved SMS message provider to retain participants for whom the SMS message does not deliver for, so they can be included in an intention to treat analysis.'
Low data completeness (64%) – Falls below typical 80% threshold. Discuss acceptability for SMI population.	This study recruited GP practices, not individual participants, under an opt out model. Data incompleteness was therefore due to GP practices encountering barriers when extracting the patient outcome data. This has already been discussed at length throughout the discussion. No changes have been made.
Low survey response (7%) – Recommend alternative fidelity methods (link click analytics, GP record review).	Thank you for raising this, which is already included in table 3):<br>'Adopt other ways to assess intervention fidelity (whilst still minimising interference with participants), e.g.: a) improve link click detection and analytics data from the links. b) collect further data from participating GP practices about impact of the study (any changes in screening consultations with the target group- ask them to note down if anyone asked about additional requirements, etc. Before/after.'
Baseline imbalance in comorbidities – Control arm had more participants with ≥1 LTC (48% vs 22%). Recommend stratified randomisation in definitive trial (noted in Table 3 – good).	We agree- as you point out, this is already suggested in table 3.
GP practice recruitment rate (27%) – Discuss potential selection bias.	Thank you for this suggestion. We have added this in the strengths and limitations section of the discussion:<br><br>'*Finally, the relatively low GP practice recruitment rate (27%) may indicate possible selection bias for a definitive trial. However, the systematic selection of participants within each GP practice using the opt out model arguably minimises the opportunity for bias, given that selection bias in GP practice research is mainly present in the characteristics of participating GP practices (compared to non-participating practices) and in the patients that GPs recruit, rather than any differences between patients registered at participating and non-participating practices (37, 38).*'
No power calculation for pilot – Acceptable but state target based on published guidance.	Already included under 'Participant eligibility and identification' section of the methods:<br><br>'Given that this pilot study aimed to assess feasibility rather than a definitive effect, no formal sample size was conducted. Instead, the

	target sample size was set based on existing guidance (a minimum of 35 participants per arm) (25, 26), with an additional 20% threshold to account for estimated opt-outs’
Single geographic area (London only) – Limits generalisability.	Thank you for raising this. Although generalisability of the sample is not important here (as it would be in a definitive trial), I understand your point about the feasibility of a larger trial and how much recruitment in London can inform recruitment across the UK more widely. I have therefore added this in the strengths and limitations section of the discussion:<br><br>‘*A more noteworthy limitation is that this study was conducted in London, which may differ to the rest of the UK in terms of GP practice engagement in research (39), affecting GP recruitment in a larger definitive trial.*’
Opt-out model – Acknowledge that participant characteristics remain unknown.	This is not relevant given that no participants opted out of this study. We therefore do know participant characteristics for all participants (Table 2).
Date of SMI diagnosis as year only – Proxy measure only.	This has already been acknowledged in the limitations section.
**Reviewer 2**	
Comments to the Author:<br>The article is overall well written but there are some areas that need improvement.	Thank you for your positive feedback and fair and considered comments. Please see our response to your specific concerns below.
Introduction:<br>The introduction lacks some detail. There is a statement that cervical cancer is almost entirely preventable, although a reference is given more detail about why it is preventable is ideally required. There is reference to screening and vaccination but it is not explicitly stated. Also the reference to vaccination needs more detail as it is not a direct vaccination for cervical cancer.	Thank you for raising this point. Clarity has been added in the first paragraph of the introduction around vaccination, screening, and why both in conjunction with each other mean that cervical cancer is preventable at a population level.
There is a considerable part of the introduction that relates to the development of a leaflet/tool (note that there is some confusion as to whether it is a leaflet or a tool or both), however it is not fully clear whether this tool was directly used to inform the SMS intervention (or the SMS intervention content). There is also reference made to the fact that the tool appears to have been widely disseminated before the trial and therefore potentially impacting on the trial.	I completely understand the confusion around leaflet vs tool. I have now referred to it as a leaflet throughout, to avoid confusion.<br><br>On reading it back, I agree that the explanation could have been clearer. We have added some connecting sentences at the end of paragraphs 4 and 5 of the Introduction to better explain the steps of how we created the intervention:<br><br>Paragraph 4: ‘*To support implementation of the leaflet in practice, we sought to identify a*

	feasible method of delivering the leaflet (via the government guidance webpage) to individuals with SMI, and to evaluate whether this could improve cervical screening attendance in this low-uptake population.'<br><br>Paragraph 5: '*We therefore developed an enhanced GP-endorsed SMS reminder for people with SMI, which included tailored behavioural content through Public and Patient Involvement (PPI) and a link to the leaflet in the form of the government guidance webpage*.'<br><br>We already noted and accounted for potential wider dissemination of the government guidance within the trial, by asking participants from both arms whether they had accessed the leaflet via the invitation letter (see the 2nd paragraph of the methods). Unfortunately, the low response rate meant that we could not assess this. We do highlight the need to assess fidelity as a priority for a definitive trial. I have added emphasis on this in the first paragraph of the discussion:<br><br>*'Alternative methods (to surveys delivered by SMS) are required to understand whether and how patients might engage with resources,* ***including outside of the intervention SMS message*** **(and the invitation letter).**'<br><br>I have also moved the order around slightly in paragraph 4 just to clarify that there has been no evaluation of the leaflet within the government guidance- only the original leaflet.
Methods:<br>Generally the methods are well laid out and detailed. However the primary outcomes as stated in the methods are not fully corresponding to what is stated in the abstract "feasibility outcomes" or results section. Namely giving detailed measures but not explicitly stating key feasibility measures such as recruitment rates, retention rates, acceptability etc.	Thank you for raising this important point. We have now ensured consistency across the abstract, methods, results, and discussion (including Table 3) in terms of the feasibility outcomes and their constituent measures. We have divided the feasibility outcomes into; retention and recruitment, data completeness, achieving comparable groups, and fidelity, and have organised the measures accordingly.
