# Supplementary material for: Behaviourally informed text message reminders to increase cervical screening attendance in people with severe mental illness: the OPTIMISE pilot randomised controlled trial
Source: BMJ Open. 2026 Sep 11;16(9):e122934. doi: 10.1136/bmjopen-2026-122934 (PMC13583816; doi:10.1136/bmjopen-2026-122934)
Supplement: Supplementary file 2 [file bmjopen-16-9-s001.docx]

Behavioural SMS reminders with link to decision tool to improve uptake of cervical screening amongst people with severe mental illness that are overdue: a pilot randomised controlled trial

Improving uptake of cervical screening in people with severe mental illness

OPTIMISE Trial

This protocol has regard for the HRA guidance and order of content

PROTOCOL VERSION NUMBER AND DATE: V7 June25

| **Amendment no.** | **Protocol version no.** | **Description of changes** | **Date of document** |
| --- | --- | --- | --- |
| 1 | Version 2 | JL- Following advice from REC committee, we will also collect data (via practice screening staff) on whether intervention participants who attend for screening have accessed the information that is part of this pilot study- this has been added as a secondary objective. Re feasibility outcome measures we have removed the number of times they click on the link- as this is unattainable. Re schedule of events- have added that iPLATO will contact the practices to ensure those who have opted out are removed before randomisation. They will inform practices of the final list and provide a unique identifier for each participant. We have also updated the sample size calculation such that the sample size is now 44 participants per arm. Updated to PPI section to reflect that a new group of women with SMI supported the team to develop the participant information sheet and amendments to the invitation letter. A link to the units website and has been added to the PIS where participants will find a lay summary at the end of the study. | 15^th^ Mar 2023 |
| 2 | Version V3 | FG- Study personnel have been updated. Have added REC reference number. Have updated outcome measures- removed HPV vaccination status and disease severity as they are poorly recorded in GP patient records. Following PPI recommendations, we have updated the consent procedures and study design to reflect that we will no longer be inviting eligible individuals to participate/opt-out in the study by letter. Instead we have sought CAG approval for iPLATO to receive the Date of Birth, NHS number and mobile phone numbers of eligible patients without consent in order to facilitate the delivery of the text message. (iPLATO will no longer be posting invitation letters and removing opt outs.) They will receive a list of eligible patients from practices, randomly assign them to the intervention arm and send out the text messages. They will also assign participants a PID number and share with practices. Practice staff, rather than iPLATO, will search GP records for participant demographic and health data, pseudonymise it and share with the data analysis members of the research team at Newcastle. Type 2 opt-outs have been added as exclusion criteria in the search of eligible patients. If patients request to opt-out after data collection (via responding via link in CS SMS reminder) we will disregard any data gathered and exclude them from the analysis. iPLATO will remove these Type 2 opt-outs from the list of eligible patients. Have added further information about factors influencing CS attendance amongst this patient group in the background to this study. | 28^th^ Feb 2024 |
| 3 | Version 4 (V4) | BN- study personnel and project timelines have been updated. The SMS text messages have been amended to minimise the cost of the study and improve the potential scalability of the intervention SMS text message. The protocol has been amended to ensure clarity that practice staff will not collect survey data relating to participants, and that this will instead be collected by iPLATO, as there were still some references to this remaining after amendment 2. Clarity has been added to state that there will be two versions of the survey: one for the control group, and another for the intervention group. The notification posters for GP practices to place in their waiting rooms and on their websites have been amended so that participants opt out via the iPLATO opt-out link to reduce the workload for GP practices. The description within the notification poster has also been amended to provide clarity that only patients with a diagnosed severe mental illness (SMI) will be involved in the study. This information has also been underlined so that patients can more easily identify themselves as eligible or not. Have also added a link to the project website in addition to a QR code for accessibility. Finally, after consultation with a GP to develop the search terms to retrieve patient data, the outcome of scheduled cervical screening appointment was removed as it was not possible to collect this information from GP records, as well as the number of times participants have been recorded as non-responders, as date of last screening was judged to be sufficient. | October 2024 |
| 4 | Version 5 (V5) | The recruitment strategy of GP practices has been amended such that as well as contacting GP practices in Lambeth through email and telephone, we will also reach out to GP practices through local networks (e.g. practice manager networks, South East London Cancer Alliance). We hope that this will improve the engagement of GP practices by increasing the relevance, credibility, and familiarity of the study. This has been added under the ‘recruitment’ section of the protocol. | Nov 2024 |
| 5 | Version 6 (V6) | The recruitment strategy has been broadened to cover South East London as opposed to Lambeth as originally specified, to facilitate recruitment. | Dec 2024 |
| 6 | Version 7 (V7) | The recruitment strategy has been broadened to cover London as opposed to South Est London specifically as originally specified, to facilitate recruitment. | Dec 2024 |

# 1. i. RESEARCH REFERENCE NUMBERS

| IRAS Number: | 323832 |
| --- | --- |
| NHS REC reference: | 23/WM/0037 |
| ISRCTN Number / Clinical trials.gov Number: | ISRCTN12558681 |

# 2. ii. RESEARCH SPONSOR

| Sponsor Name: | The University of Newcastle Upon Tyne |
| --- | --- |
| Sponsor Reference: | 202223 23 Lecouturier |

# 3. iii. RESEARCH FUNDER

| FUNDER(S) | FINANCIAL AND NON FINANCIALSUPPORT GIVEN |
| --- | --- |
| National Institute for Health Research Policy Research Unit in Behavioural Science (project reference PR-PRU-1217-20501). | £50,000 research, statistician, and health economist costs |
| National Institute for Health Research Policy Research Unit in Cancer awareness, screening and early diagnosis?? | Staff time and contribution to NHS support costs |

# 4. iv. SIGNATURE PAGE

The undersigned confirm that the following protocol has been agreed and accepted. The Chief Investigator agrees to conduct the trial in compliance with the approved protocol and will adhere to the Research Governance Framework, Good Clinical Practice (GCP) guidelines, the relevant Standard Operating Procedures and other regulatory requirements as applicable.

I agree to ensure that the confidential information contained in this document will not be used for any other purpose other than the evaluation or conduct of the investigation without the prior written consent of the Sponsor.

Representative of the Research Sponsor

| Name: (print) | Susanne Lewis | | |
| --- | --- | --- | --- |
| Position: | Head of Faculty Research | | |
| Signature: |  | Date: |  |

Chief Investigator

| Name: (print) | Beth Nichol | | |
| --- | --- | --- | --- |
| Signature: |  | Date: |  |

Statistician

| Name: (print) | Andy Bryant | | |
| --- | --- | --- | --- |
| Position: | Research Associate | | |
| Signature: |  | Date: |  |

Trial Coordinators and Data manager

| Name:(print) | Beth Nichol | | |
| --- | --- | --- | --- |
| Position: | Research Assistant | | |
| Signature: |  | Date: |  |

# 5. v. KEY TRIAL CONTACTS

| Chief Investigator | Beth Nichol  Research Assistant  Population Health Sciences  Newcastle University  Baddiley Clark Building  Richardson Road |
| --- | --- |
| Trial Co-ordinators | Beth Nichol  Research Assistant  Population Health Sciences  Newcastle University  Baddiley Clark Building  Richardson Road  Aikaterini Grimani  Research Associate  Warwick University |
| Sponsor | Susanne Lewis  Head of Faculty Research  Faculty of Medical Sciences  Newcastle University  NE24HH |
| Funder(s) | NIHR Policy Research Unit in Behavioural Science  Newcastle University |
| Key Protocol Contributors (contact details in appendix) | Ivo Vlaev, Professor of Behavioural Science, Warwick University  Elizabeth Barley, Professor of Mental Health Sciences and Nursing, University of Surrey  Josephine Ruwende, Screening and Immunisation Lead (Cancer Screening) NHS England and Improvement  Christian Von Wagner, Reader, PRU in Cancer awareness, screening and early diagnosis, UCL  Martin Nemec, Research Associate, PRU in Cancer Awareness, UCL  Stephen Duffy, Director of PRU in Cancer Awareness, Queen’s Medical University London  David Osborn, Professor of Psychiatric Epidemiology; PRU Mental Health, PRU Behavioural Science, UCL  Mark Ashworth, Professor in Primary Care, KCL  Maggie Luck (NHS England),  Charlotte Klass, Public Health Practitioner, Kings College, London  Luke Vale, Professor of Health Economics, Newcastle University  Caroline Kemp, PPI representative |
| Statistician | Andy Bryant, Research Associate, Newcastle |
| Committee | Project management group |

# 6. vi. TRIAL SUMMARY

| Trial Title | Behavioural SMS reminders with link to decision tool to improve uptake of cervical screening (CS) amongst people with severe mental illness (SMI) that are overdue: a pilot randomised controlled trial | |
| --- | --- | --- |
| Acronym | OPTIMISE | |
| Summary of Trial Design | Pilot Individual randomised controlled trial with model based economic evaluation to identify the target difference for subsequent definitive RCT. | |
| Summary of Participant Population | All women (people with a cervix) with SMI between 24- 64 years who are classed as non-responders (have not had a CS during the age-appropriate interval i.e. within the previous 3.5 years for women aged 24-49 within the previous 5.5 years for women aged 50-64). | |
| Planned Sample Size | 88 (44 per arm). | |
| Planned Number of Sites | Up to 30 GP practices in London | |
| Intervention Duration | One SMS reminder | |
| Follow Up Duration | 18 weeks | |
| Planned Trial Period | November 2024 to June 2025 |  |
|  | Objectives | Outcome Measures |
| Primary | To assess the feasibility of participant identification and implementation processes | Feasibility measures: number of eligible patients, number excluded in screening and reasons for exclusion, Number of potential participants that dissent/opt-out of the study,  Number of participants for which mobile number is missing/invalid, number of texts delivered, number of times links opened. |
| Secondary | To rehearse data collection and data analyses for effectiveness and economic evaluation and refine for subsequent definitive trial  Estimate the minimum level of additional effectiveness of enhanced SMS cervical screening reminders that would be cost-effective  iPLATO tocollect basic information on whether intervention participants who attend for screening accessed the intervention information. | CS status of each participant 18-week post intervention (CS appointmentattended)    Costs of enhanced and usual text messages  GP resource use/research activity |

# 7. vii. GLOSSARY OF ABBREVIATIONS

BS	Behavioural Science

CI	Chief Investigator

CRF	Case Report Form

CS	Cervical Screening

CSAS	Cervical Screening Administration Service

DSA	Data Sharing Agreement

DNA	Did not attend

GCP	Good Clinical Practice

IMD	Index of Multiple Deprivation

ISF	Investigator Site File (This forms part of the TMF)

ISRCTN	International Standard Randomised Controlled Trials Number

NCL	Newcastle University

NHS R&D	National Health Service Research & Development

PI	Principal Investigator

PIS	Participant Information Sheet

PRU	Policy Research Unit

RCT	Randomised Control Trial

REC	Research Ethics Committee

SAE	Serious Adverse Event

SMI	Severe Mental Illness

TMG	Trial Management Group

# 8. viii. CONTENTS

[1.	i. RESEARCH REFERENCE NUMBERS	3](#_Toc197001962)

[2.	ii. RESEARCH SPONSOR	3](#_Toc197001963)

[3.	iii. RESEARCH FUNDER	3](#_Toc197001964)

[4.	iv. SIGNATURE PAGE	5](#_Toc197001965)

[5.	v. KEY TRIAL CONTACTS	6](#_Toc197001966)

[6.	vi. TRIAL SUMMARY	7](#_Toc197001967)

[7.	vii. GLOSSARY OF ABBREVIATIONS	7](#_Toc197001968)

[8.	viii. CONTENTS	8](#_Toc197001969)

[1.	BACKGROUND	12](#_Toc197001970)

[2.	RATIONALE	13](#_Toc197001971)

[2.1 Assessment and management of risk	13](#_Toc197001972)

[3.	OBJECTIVES AND OUTCOME MEASURES/ENDPOINTS	14](#_Toc197001973)

[3.1	Primary objective	14](#_Toc197001974)

[3.2	Secondary objectives	14](#_Toc197001975)

[3.3	Outcome measures/endpoints	14](#_Toc197001976)

[3.1.1.	Primary feasibility parameters (outcome measures)	14](#_Toc197001977)

[3.1.2.	Secondary feasibility parameters (effectiveness and economic outcome measures)	14](#_Toc197001978)

[4.	TRIAL DESIGN	16](#_Toc197001979)

[5.	STUDY SETTING	17](#_Toc197001980)

[6.	ELIGIBILITY CRITERIA	17](#_Toc197001981)

[7.	TRIAL PROCEDURES	17](#_Toc197001982)

[7.1	Recruitment	17](#_Toc197001983)

[**7.1.1 Participant identification**	17](#_Toc197001984)

[7.2 Consent	17](#_Toc197001985)

[7.3	Randomisation	18](#_Toc197001986)

[7.4	Blinding	18](#_Toc197001987)

[7.5	Trial assessments	18](#_Toc197001988)

[7.5.1 Feasibility assessments	18](#_Toc197001989)

[7.5.2 Future trial effectiveness assessments	19](#_Toc197001990)

[7.6 Schedule of Events	19](#_Toc197001991)

[7.7	Withdrawal criteria	19](#_Toc197001992)

[7.8	End of trial	20](#_Toc197001993)

[8.	TRIAL INTERVENTION	20](#_Toc197001994)

[8.1 Name and description of intervention	20](#_Toc197001995)

[8.3 Known Risks	21](#_Toc197001996)

[8.5 Assessment of Compliance	21](#_Toc197001997)

[9.	SAFETY REPORTING	21](#_Toc197001998)

[(Not applicable for this study)	21](#_Toc197001999)

[10.	STATISTICAL CONSIDERATIONS	21](#_Toc197002000)

[10.1 Analysis Population	21](#_Toc197002001)

[10.2 Statistical Analyses	21](#_Toc197002002)

[10.2.1 Analysis of the Primary Outcome Measure	21](#_Toc197002003)

[10.2.2 Analysis of Secondary Outcome Measures	21](#_Toc197002004)

[10.2.3 Interim Analyses and Criteria for the Premature Termination of the Trial	21](#_Toc197002005)

[10.3 Statistical Size Calculations	21](#_Toc197002006)

[10.3.1.	Sample size calculation	22](#_Toc197002007)

[10.3.2	Planned recruitment rate	22](#_Toc197002008)

[10.3.3	Procedure(s) to account for missing or spurious data	22](#_Toc197002009)

[11.	DATA HANDLING	22](#_Toc197002010)

[11.1	Data collection tools and source document identification	22](#_Toc197002011)

[11.2	Data handling and record keeping	22](#_Toc197002012)

[11.3	Access to Data	23](#_Toc197002013)

[12.	MONITORING, AUDIT & INSPECTION	23](#_Toc197002014)

[13.	ETHICAL AND REGULATORY CONSIDERATIONS	23](#_Toc197002015)

[13.1	Research Ethics Committee (REC) review & reports	23](#_Toc197002016)

[13.2	Peer review	23](#_Toc197002017)

[13.3	Public and Patient Involvement	24](#_Toc197002018)

[13.3.1 Design of research	24](#_Toc197002019)

[13.3.2 Management and dissemination of research	24](#_Toc197002020)

[13.4	Regulatory Compliance	24](#_Toc197002021)

[13.5	Protocol compliance	24](#_Toc197002022)

[13.6	Notification of Serious Breaches to GCP and/or the protocol	24](#_Toc197002023)

[13.7	Data protection and patient confidentiality	25](#_Toc197002024)

[13.8	Financial and other competing interests for the chief investigator at each site and committee members for the overall trial management	25](#_Toc197002025)

[13.9	Indemnity	25](#_Toc197002026)

[13.10	Amendments	25](#_Toc197002027)

[13.11	Access to the final trial dataset	25](#_Toc197002028)

[14.	14 DISSEMINATION POLICY	25](#_Toc197002029)

[14.1	Dissemination policy	25](#_Toc197002030)

[14.2	Authorship eligibility guidelines and any intended use of professional writers	26](#_Toc197002031)

[15.	REFERENCES	26](#_Toc197002032)

[16.	APPENDICIES	28](#_Toc197002033)

[17.	Appendix 1 – Amendment History	28](#_Toc197002034)

[18.	Appendix 2: Statistical Analysis Plan	30](#_Toc197002035)

# 1. BACKGROUND

People with a diagnosed severe mental illness (SMI) including schizophrenia, bipolar affective disorder and other psychoses, are more likely than the general population to experience poor physical health and to die prematurely [1,2]. Cancer is the leading cause of premature mortality among people with SMI ^[1]^[2]. Evidence suggests that people with SMI are 2.1 times more likely to die from cancer before the age of 75, compared to people without SMI. Mortality rates in people with SMI are continuing to rise worldwide and in England by 5%–10% every 2 years due to low cancer screening uptake [3,4]. In particular, cervical cancer is the second most common cancer among women 15-44 years ([ONS 2017](https://www.ons.gov.uk/peoplepopulationandcommunity/healthandsocialcare/conditionsanddiseases/bulletins/cancerregistrationstatisticsengland/2017#the-three-most-common-cancers-vary-by-sex-and-age-group)) [5]. Whilst rates of cervical cancer nationally have reduced since the introduction of the UK cervical screening programme in 1988, analyses from 2018 found that people with SMI, particularly women aged 45-64, were 20% more likely not to have participated cervical screening within the recommended time period than people without SMI [1].

The current literature from both England and worldwide supports the evidence that people with SMI tend to have low participation rates in cervical screening and are at a higher risk of cervical neoplasia [4,6,7]. Additional relevant studies highlight that participation in cervical screening is notably low among individuals with schizophrenia [6,8] compared to those with other psychoses and bipolar disorder, those living in deprived areas (with SMI) [6,8], and those of Asian ethnicity [6]. Ethnic minorities without SMI were less likely to participate compared to White individuals with SMI, highlighting significant ethnic disparities. There are complex interactions between SMI, physical comorbidity, and cancer screening behaviours. Smoking found to be associated with lower participation in cervical screening, but it did not worsen screening among those with SMI. Moreover, BMI was not found to affect participation among those without SMI. However, slightly lower participation was observed among those with SMI and a BMI < 30 [6]. Two studies found a positive correlation between the level of physical comorbidity and the likelihood of receiving a cervical screening among people with SMI [8,9]. A study conducted in Canada [10] found that SMI combined with other long-term illnesses like inflammatory bowel disease (IBD), multiple sclerosis (MS), and rheumatoid arthritis (RA), can affect cervical screening differently depending on the specific disease. Individuals with SMI and either MS or RA were more likely to participate in cervical screening. However, the frequency of cervical screening was lower for individuals with SMI and IBD [10]. This reflects significant barriers to both diagnosis and treatment of cervical cancer in the SMI population which need to be addressed.

In England, all women and people with a cervix aged between 25-64 are eligible for cervical screening. Those aged between 24.5-49 receive an invitation every three years and those aged 50-64 every five years [11]. The Cervical Screening Administration Service (CSAS) sends out call and recall letters to eligible patients for cervical screening on behalf of GP practices. Before sending out invitations the CSAS provides GP practices with lists of patients due to be invited for screening, known as Prior Notification Lists (PNL). General practices review these lists weekly to check eligibility for screening. When the new [NHS Cervical Screening Management System is launched in 2022 these lists will be managed by practice staff via this system.](https://digital.nhs.uk/services/screening-services/national-cervical-screening/new-cervical-screening-management-system) Patients receive a letter from the CSAS inviting them to contact their local GP practice to arrange an appointment with a nurse to have cervical screening. Women that do not arrange to have a cervical screening within the recommended time frame for their age group are classed as ‘non-responders’ (See Figure 1).

Figure 1- **Timeline of the call and recall pathway for cervical screening and the point at which patients become classed as non-responders**.

12 days after reminder letter, 2^nd^ SMS reminder sent

12 days after initial invitation, 1^st^ SMS reminder sent

PNL-Prior Notification List, NTTD- Next Test Due Date [Cervical screening: guidance for call and recall administration best practice - GOV.UK (www.gov.uk)](https://eur03.safelinks.protection.outlook.com/?url=https%3A%2F%2Fwww.gov.uk%2Fgovernment%2Fpublications%2Fcervical-screening-call-and-recall-administration-best-practice%2Fcervical-screening-call-and-recall%23pnl&data=04%7C01%7CFiona.Graham%40newcastle.ac.uk%7Cc9fa4fba9dac4d354fb508d9e00f2b5c%7C9c5012c9b61644c2a91766814fbe3e87%7C1%7C0%7C637787178026726515%7CUnknown%7CTWFpbGZsb3d8eyJWIjoiMC4wLjAwMDAiLCJQIjoiV2luMzIiLCJBTiI6Ik1haWwiLCJXVCI6Mn0%3D%7C3000&sdata=h4Hm5%2BOcZrpi14fJtHEPYcXyrG6xOxzEzpWnJ1oP54E%3D&reserved=0)

A trial examining the impact of text message reminders and their context on cervical screening rates [12] was so successful that SMS have now been incorporated into a London-wide screening SMS campaign. Public Health England (PHE) reported that after six months cervical screening attendance increased by 4.8%, which is the equivalent of 13,400 more women being screened at 18 weeks. However, it is unclear whether such text message reminders support women with SMI.

To improve cervical screening rates among women with SMI, an informed choice tool (information leaflet) has been developed by mental health service users and healthcare professionals [13]. It includes tips and a checklist of factors that might make the test difficult that patients can share with the nurse. The tool has been converted into a webpage available on the government/PHE website- ‘Cervical screening- support for people who feel anxious about attending’ [14]. It is also available as pdf leaflet on Jo’s Cervical Cancer Trust website [15] and as an animation on Youtube [16]. Ultimately, we propose to determine whether including a message framed around social norms with a link to this tool in a multimedia SMS cervical screening reminder (enhanced screening reminder) will increase CS uptake by women with SMI that are overdue (classed as non-responders).

# 2. RATIONALE

Patient and carers of women with SMI groups have informed us that the decision tool it an excellent resource and something they, or their family, would find very helpful. They are currently unaware of these materials and how they might be accessed. They have also reported that they find text message reminders useful. Text message reminders are a relatively low-cost intervention which have already been found to increase uptake of cervical screening. By tailoring the message for women who find it difficult to attend and providing supportive material may help to address the current disparity in cervical screening uptake by women with SMI.

## 2.1 Assessment and management of risk

This trial is categorised as Type A, No higher than the risk of standard medical care. SMS reminders about cervical screening are routinely used by GP practices to encourage women to make aIRASn appointment. The information to be incorporated into the SMS reminder as part of the intervention is publicly available on the Jo’s Cancer Trust website and on YouTube and therefore unlikely pose any risk to the participant.

# 3. OBJECTIVES AND OUTCOME MEASURES/ENDPOINTS

To establish the feasibility of conducting a randomized controlled trial (RCT) that compares enhanced SMS cervical screening reminders with usual SMS reminders to increase screening uptake among people with SMI that are overdue (did not attend (DNA)/non-responders).

Objectives:

- To assess the feasibility of participant identification and implementation processes
- To rehearse data collection and data analyses for effectiveness and economic evaluation and refine for subsequent definitive trial
- Estimate the minimum level of additional effectiveness of enhanced SMS cervical screening reminders that would be cost-effective

## 3.1 Primary objective

- To assess the feasibility of participant identification and implementation processes

## 3.2 Secondary objectives

- To rehearse data collection and data analyses for effectiveness and economic evaluation and refine for subsequent definitive trial
- Estimate the minimum level of additional effectiveness of enhanced SMS cervical screening reminders that would be cost-effective
- Collect data (via SMS text message provider iPLATO) on whether intervention participants who attend for screening have accessed the information that is part of this pilot study, either through the intervention SMS text message or the invitation letter that is sent to all participants as part of usual care

## 3.3 Outcome measures/endpoints

3.1.1. Primary feasibility parameters (outcome measures)
- Number of eligible participants per practice
- Number of participants who opt out of the study
- Number of invalid phone numbers
- Number of missing phone numbers
- Number of participants where primary outcome data is missing
- SMS delivery status (provided by iPLATO)
- Number of participants who access the tool/ click on the link in the text
  3.1.2. Secondary feasibility parameters (effectiveness and economic outcome measures)
- Proportion of women in each arm who attended a CS appointment within 18 weeks of the SMS reminder being sent. Age, gender, ethnicity and index of multiple deprivation (IMD) decile, date of last CS and CS test result, will be measured as co-variates.
- Resource use/costs incurred

**3.3	Table of outcomes**

| Objectives | Outcome Measures |
| --- | --- |
| **Primary objective**  To assess the feasibility of participant identification and implementation processes | - Participant characteristics: Age, gender, ethnicity and index of multiple deprivation (IMD) decile, date of SMI diagnosis, one or more comorbidities (Y/N), whether they attended a CS appointment (Y/N) within 18 weeks of SMS, date of last CS appointment). - number of eligible participants per practice - number of participants that opt out of the study - number of invalid phone numbers - number of missing phone numbers - number of participants where primary outcome data is missing - SMS delivery status (provided by iPLATO) - Number of participants who access the tool/ click on the link in the text |
| **Secondary Objectives** To rehearse data collection and data analyses for effectiveness and economic evaluation and refine for subsequent definitive trial  Estimate the minimum level of additional effectiveness of enhanced SMS cervical screening reminders that would be cost-effective  Collect data (viaSMS text message provider iPLATO) on whether intervention participants who attend for screening have accessed the information | - Proportion of women in each arm who attend a CS appointment within 18 weeks of the SMS reminder being sent. - Resource use/costs incurred |

# 4. TRIAL DESIGN

Pilot randomised controlled trial (parallel design) to rehearse and test processes for a full-scale trial.

**Quantitative data analyses**:

- Screening rates % (eligible, n/ screened)
- Number of patients that opt-out (n)
- Numbers that dissent

Numbers that did not receive allocated intervention/ SMS delivery status

- Uptake at 18 weeks (attendance at CS appointment)
- Whether intervention participants who attend for screening

accessed the information

To intervention (n= )

Patients receive text reminder with link to decision tool (webpage) and animation

Patients screened for eligibility by GP (n= )

Excluded (n=)

Reasons (…)

Not meeting inclusion criteria (n=)

Randomized (n= )

To control (n= )

Patients receive usual text reminder

Patients identified in computer search by practice staff (n=)

GP practices provide iPLATO a list of eligible patients-people with SMI overdue cervical screening (non-responders). iPLATO then randomly allocate patients to each arm and assigns PID numbers.

At 18 weeks, practice staff run search of GP records for participant health information and whether an CS appointment has been attended for screening (Y/N). All participants are sent a link to a survey (the exact survey depends on whether they are assigned to the intervention or control group). Practice staff complete GP research activity proforma.

.

**Inclusion criteria**

- Aged between 24-64 years
- Diagnosed with SMI
- Classed as non-responders on CSAS list sent to GP practices.
- Registered with a participating practice

**Exclusion criteria**

- Those the general practitioner considers it inappropriate to approach.
- Ineligible for screening
- Those that have dissented to have their data used for research purposes (Type 2 opt-outs).

**Health economic analysis**:

Cost of enhanced SMS reminder compare with usual (proforma), time taken for practices to identify non-responders with SMI and other research activities (proforma)

Screened

Assessment

# 5. STUDY SETTING

This is a multisite trial involving GP practices within PCNs/federations in London where SMI prevalence is relatively high compared to the national average.

# 6. ELIGIBILITY CRITERIA

All women with SMI between 24- 64 years that are classed as non-responders (have not had a CS during the age-appropriate interval i.e. within the previous 3.5 years for women aged 24-49 within the previous 5.5 years for women aged 50-64).

6.1	Inclusion criteria

- Aged between 24-64 years
- Diagnosed with SMI
- Classed as non-responders (6 months or more overdue).
- Registered with a participating practice

6.2	Exclusion criteria

- Those judged inappropriate to approach at a search level (individuals who have declined screening in the last 12 months and individuals who have ceased screening invitations from the CSAS).
- Ineligible for screening- e.g. pregnant individuals, individuals receiving palliative care.
- Type 1 opt outs (opted out of receiving SMS messages)
- Type 2 objectors/opt outs (a registered patient who has indicated that they do not want their personal information being shared by GP practices for purposes other than their own direct care (including research)
- Those the general practitioner considers it inappropriate to approach (at the discretion of the GP practices)

# 7. TRIAL PROCEDURES

## 7.1 Recruitment

We will use purposive sampling to recruit a range of practices in London that vary with regards to patient population size, research experience, cervical screening rates and numbers of women with SMI. Based on the available Quality Outcomes Framework data we can identify and approach practices where uptake of CS is low in women with SMI. We have a list of practices iPLATO have data sharing processing with and deliver their SMS. Our contact in the South East London Integrated Care system will provide us with contact details of practices that we will invite by email and telephone call. To increase engagement of GP practices, we will also reach out to GP practices in London through local networks (e.g. practice manager networks and the South East London and North East London Cancer Alliances); facilitated by key contacts across South East and North East London.

### **7.1.1 Participant identification**

General Practitioners/ practice managers in participating practices, will screen patient lists using the read codes for SMI and non-responders. Practices will identify lists of eligible participants and send their details (name, NHS number and mobile phone number) to iPLATO.

## 7.2 Consent

Practices that agree to participate in this study will update their data processing agreements with iPLATO to enable them to share the NHS numbers of eligible patients in order to facilitate the delivery of the text messages as part of the study. This study will be covered by Section 251, patients are not required to give informed consent before their confidential identifiable information (name, NHS numbers and mobile phone numbers) are provided to iPLATO. Participating practices will agree for patient notification posters to be placed in their waiting rooms and on their website so patients are aware about the study and can dissent for their data being used in this study if they wish. Patients that have indicated their wish to opt-out of having their personal confidential information shared for research purposes (Type 2 objectors/opt-outs) will be removed from the list of eligible participants by the practice staff at the screening stage, and by iPLATO if anyone dissents opts-out of the study after the initial screening phase.

## 7.3 Randomisation

The randomisation process will be conducted by iPLATO using a random number generator. Participants will be allocated intervention group 1:1.

For the purpose of the study iPLATO will add a unique identifier and a ’group randomised to’ code to the data set before it is shared with the practices and subsequently with the research team for analyses.

## 7.4 Blinding

Patients and GP staff will be blinded to the group allocation.

## 7.5 Trial assessments

### 7.5.1 Feasibility assessments

To assess the feasibility of the study and data collection processes the following data collected on proforma completed by iPLATO:

- Number of participants that opt out of the study
- Number of invalid phone numbers
- Number of missing phone numbers
- Number of participants where CS status is missing
- SMS delivery status (provided by iPLATO)
- Number of participants who click on the link to website/animation
- Costs incurred (proforma completed by iPLATO regarding postage and SMS costs)

To assess the feasibility of general practice resource use, practice managers will be asked consulted about resources required to undertake the various research activities as part of this study including: identify eligible participants (complete detailed screening logs), collecting information about participants through searches of GP records at 18 weeks, pseudonymising and sharing data collected with the research team at NCL. Details of these conversations will be recorded on a proforma completed by the research team at NCL.

iPLATO staff will be consulted regarding the costs incurred from participating in this research study (for example, updating data processing agreements with practices, randomizing the list of patients, assigning PID numbers etc). These estimates will inform costings of a potential future definitive trial. Conversations with iPLATO staff will also explore costs incurred by the intervention itself, the enhanced text message reminder, to inform the health economics evaluation. (For example, the costs involved with setting up/ changing the tech systems to send out the enhanced text message reminders). They will also be asked to provide insights into the costs they anticipate if the enhanced messages were to replace the existing text messages at scale. Details of these conversations will be recorded on a proforma completed by the research team at NCL.

### 7.5.2 Future trial effectiveness assessments

iPLATO will randomise and send those in the intervention arm the enhanced text message reminder and those in the control group the standard reminder. After 18 weeks, GP staff will run a search of their clinical systems and complete a case report form for each study participant collecting the following information:

- patient demographics data (e.g., age, gender, ethnicity, first part of postcode to derive IMD)
- patient characteristics (e.g., date of SMI diagnosis, comorbidities, date of last screening appointment
- Screening appointment attended following SMS reminder(Y/N)

This data together with allocation status (control/intervention) will be pseudonymised and shared with the data analysis team at Newcastle University (each participant will have a PID rather than a personal identifier). The PID key will be retained by iPLATO and the participating practice and destroyed once the data has been analysed.

## 7.6 Schedule of Events

1. NCL provides participating GP practices with search terms/code to identify patients with SMI that are non-responders.
2. GPs review the list to determine whether patients are eligible to participate in the study
3. GP practices provide iPLATO with a list of eligible patients (NHS numbers, Date of Birth) with SMI that are overdue cervical screening (non-responders)
4. iPLATO randomises patients to receive either a control text reminder (no link) or intervention enhanced text reminder (with link). (They use the NHS number to access clinical systems to send the text message). At the end of the text message there will be a link to a GP privacy notice and a link to enable them opt-out of this specific study.
5. iPLATO will receive and remove any study specific opt-outs from the list of participants.
6. iPLATO will collect SMS feasibility measures and save with DOB, NHS and PID numbers on a participant case report form and securely share it with GP practices.
7. After 18 weeks, iPLATO will send all participants a SMS with link to survey to ask whether they accessed the text from their invitation letter (both control and intervention groups) information materials that are the focus of this study (intervention group only), which ones (animation and/or textual, intervention group only) and whether they watched/read the information. iPLATO receives survey responses, ensures they are then share with the data analysis team at Newcastle University).
8. After 18 weeks, practice staff will search the GP systems for the agreed patient data and add it to the participant case report form.
9. They will remove the NHS numbers and DOBs from the participant case report form and securely share the pseudonymised dataset with the team at NCL.
10. GP staff provide data analysis team at Newcastle University with pseudonymised dataset.
11. GP staff and iPLATO will be consulted regarding the amount of time they have spent on the research activities as part of the economic and feasibility assessment of this study.

## 7.7 Withdrawal criteria

Participants have the right to dissent for their information being used as part of this trial at any time without having to give a reason. Type 2 objectors will be removed from the dataset by practice staff when searching for eligible patients and when searching for participant information at after 18 weeks. iPlato will remove any participants that ‘opt-out’ specifically to this study via the link in the SMS reminder.

The Investigator may discontinue a participant from the trial at any time if the Investigator considers it necessary for any reason including:

- Participant withdrawal of consent
- GPs discretion that it is in the best interest of the participant to withdraw
- An adverse event that requires discontinuation of the trial intervention or renders the participant unable to continue in the trial
- Termination of the pilot trial by the sponsor

Should a patient no longer be eligible for a cervical screening, due to changes in medical circumstances, it will be the GP’s responsibility to contact that trial co-ordinator and withdraw them from the study.

As this is a pilot/feasibility study, patients withdrawn from the study will not be replaced but reasons for withdrawal will be recorded wherever possible

## 7.8 End of trial

The trial will end when the data for the last participant is retrieved from GP records by practice staff and the survey data has been collected from iPLATO.

# 8. TRIAL INTERVENTION

## 8.1 Name and description of intervention

An SMS has been developed with wording framed around social norms (informed with the input of mental health service users and their carers). It will include a link to the leaflet and the animation explaining the leaflet:

Text reminder ([Practice Name]: You have not had your cervical screening test. Please call [GP number] to book. Info at www.gov.uk/phe/cervical-screening-leaflet. Other people have found this animation *and* guidance [INSERT LINK] helpful.  It provides extra support with the test including tips about what you can do before and during the appointment that may make the test easier for you. Please ignore if you have already booked. Privacy notice: mygp.me/CS-privacy.

*[Practice Name]: You have not had your cervical screening test. Please call [GP number] to book. Info at* [*https://mygp.care/CSLeaflet*](https://eur03.safelinks.protection.outlook.com/?url=https%3A%2F%2Fmygp.care%2FCSLeaflet&data=05%7C02%7Cbeth.nichol%40newcastle.ac.uk%7C0db2448207754a536f4908dce14396bd%7C9c5012c9b61644c2a91766814fbe3e87%7C1%7C0%7C638632928054964116%7CUnknown%7CTWFpbGZsb3d8eyJWIjoiMC4wLjAwMDAiLCJQIjoiV2luMzIiLCJBTiI6Ik1haWwiLCJXVCI6Mn0%3D%7C0%7C%7C%7C&sdata=9SvQRo3n6ZZEGd%2FyaXWXEQdk7Db2RwzUkAoMfUz%2F9L4%3D&reserved=0)  *Worried about attending? Other people have found this animation [link to animation] and guidance [link to guidance] helpful for tips and support. Privacy notice:* [*mygp.me/CS-privacy*](https://eur03.safelinks.protection.outlook.com/?url=http%3A%2F%2Fmygp.me%2FCS-privacy&data=05%7C02%7Cbeth.nichol%40newcastle.ac.uk%7C0db2448207754a536f4908dce14396bd%7C9c5012c9b61644c2a91766814fbe3e87%7C1%7C0%7C638632928055052968%7CUnknown%7CTWFpbGZsb3d8eyJWIjoiMC4wLjAwMDAiLCJQIjoiV2luMzIiLCJBTiI6Ik1haWwiLCJXVCI6Mn0%3D%7C0%7C%7C%7C&sdata=lm%2B6hfT%2FfkYpOVcPHjm3Kos%2FV77cVrpOlP6N7vaaUuQ%3D&reserved=0) *This is part of a research study, click here for more info or to opt out: [opt-out link]*

Control arm

Patients allocated the control group will receive the usual standard SMS reminder, although ‘Please ignore if you have already booked’ will be removed from both the intervention and control texts as all participants will be non-responders:

*[Practice Name]: You have not had your cervical screening test. Please call [GP number] to book. Info at*  [*https://mygp.care/CSLeaflet*](https://eur03.safelinks.protection.outlook.com/?url=https%3A%2F%2Fmygp.care%2FCSLeaflet&data=05%7C02%7Cbeth.nichol%40newcastle.ac.uk%7C0db2448207754a536f4908dce14396bd%7C9c5012c9b61644c2a91766814fbe3e87%7C1%7C0%7C638632928054964116%7CUnknown%7CTWFpbGZsb3d8eyJWIjoiMC4wLjAwMDAiLCJQIjoiV2luMzIiLCJBTiI6Ik1haWwiLCJXVCI6Mn0%3D%7C0%7C%7C%7C&sdata=9SvQRo3n6ZZEGd%2FyaXWXEQdk7Db2RwzUkAoMfUz%2F9L4%3D&reserved=0) *Privacy notice:* [*mygp.me/CS-privacy*](https://eur03.safelinks.protection.outlook.com/?url=http%3A%2F%2Fmygp.me%2FCS-privacy&data=05%7C02%7Cbeth.nichol%40newcastle.ac.uk%7C0db2448207754a536f4908dce14396bd%7C9c5012c9b61644c2a91766814fbe3e87%7C1%7C0%7C638632928054987507%7CUnknown%7CTWFpbGZsb3d8eyJWIjoiMC4wLjAwMDAiLCJQIjoiV2luMzIiLCJBTiI6Ik1haWwiLCJXVCI6Mn0%3D%7C0%7C%7C%7C&sdata=BZ%2Fjae95Yt8lL32n7bSv%2BoexT2489pc1C25XJ4iJ3qM%3D&reserved=0) *This is part of a research study, click here for more info or to opt out: [opt-out link]*

”

To control for any effect the timing of the intervention might have, all SMS will be sent at the same day/time via iPLATO patient care messaging.

## 8.3 Known Risks

No known risks.

## 8.5 Assessment of Compliance

Number of text messages delivered and whether the person clicks on the link to information will be recorded by iPLATO.

# 9. SAFETY REPORTING

## (Not applicable for this study)

# 10. STATISTICAL CONSIDERATIONS

As this is a pilot trial, the main analyses will be descriptive.

## 10.1 Analysis Population

Any participant randomised into the trial, regardless of whether they received intervention or not will be included in the descriptive analyses. Definitive trial would follow an intention to treat analysis.

## 10.2 Statistical Analyses

### 10.2.1 Analysis of the Primary Outcome Measure

Feasibility parameters (screening rates, recruitment rates, missing data) will be presented in simple descriptive tables presenting percentages, means and standard deviations for each arm of the study.

### 10.2.2 Analysis of Secondary Outcome Measures

Analyses of the primary outcome measure that would be conducted in a definitive trial will be rehearsed to look for trends in the data. Logistic regression will be performed to assess the association between trial arms and uptake before and after adjusting for baseline characteristic on an intention-to-treat and per protocol basis. Absolute rates of uptake will be reported in addition to the odds ratios from logistic regression. These analyses will be performed using R/Python.

### 10.2.3 Interim Analyses and Criteria for the Premature Termination of the Trial

There will be no interim analysis for this pilot trial.

As this is a feasibility trial it is very unlikely that this this study will be prematurely terminated. Instances such as a very low recruitment rate (<1 patient a month) or a major outbreak of Covid-19 may make it impossible to continue. The study sponsor (Newcastle University) has the ultimate authority to stop or modify the trial.

## 10.3 Statistical Size Calculations

The trial analyses will follow a pre-defined Statistical Analysis Plan (SAP, see Appendix 2), a version-controlled document written by the statisticians, signed by the CI.

### 10.3.1. Sample size calculation

The candidate primary outcome measure for a future definitive trial design will be CS uptake (the proportion of women that attend a CS appointment within 18 weeks of receiving the SMS). The assumed uptake for the control arm would be 64% (based on coverage data from Lambeth, a borough in South East London- percentage of by women with SMI that have had a CS within the appropriate timeframe). We anticipate that the uptake in the intervention arm would be 5 percentage points higher (i.e. 69%). We need to compensate for those who opt out of the study. However, we only have data on the opt out rate in a cervical screening study of women in London (Huf et al. 2020) and not specifically women with SMI. The general population had a very low opt-out rate. If we work on the assumption that 20% women with SMI will opt-out/missing mobile numbers, this leaves a sample size of 88 (44 per arm).

### 10.3.2 Planned recruitment rate

- The mean number of eligible women (aged 24-64 with SMI who are non-attendees) per practice in Lambeth (a borough in South East London) is 14, (range 4-44)
- We anticipate screen failure rate and opt-out rate to be around 20%
- We anticipate that we would need to recruit up to 30 practices to achieve a sample size of 88.

### 10.3.3 Procedure(s) to account for missing or spurious data

- Reasons for missing data will be recorded where possible

# 11. DATA HANDLING

## 11.1 Data collection tools and source document identification

Data collection tools:

- Electronic screening logs will be used by practices the numbers of eligible participants and reasons why individuals are ineligible.
- iPLATO will record the number of patients that dissent to for their data being used in the study on a recruitment log.
- Electronic case report form (eCRF) will be created for each individual participant to collect feasibility outcome data, demographic information and CS appointment attendance at 18 weeks post intervention. (Only the data required by the protocol are captured in the CRF)
- Resource use proformas (completed through discussions with iPLATO staff)
- Research activity proformas (completed through discussions with practice staff)
- Record whether intervention participants accessed intervention information survey (completed bythe participant via a link hosted and sent by iPLATO)

## 11.2 Data handling and record keeping

Data processing agreements (DPA) will be in place between participating practices and the third party iPLATO. iPLATO is a contractor for NHS England and also conduct projects in collaboration with the NHS. DPAs will allow iPLATO to receive patient identifiable information (NHS numbers, DOB and mobile numbers) for the purposes of facilitating the delivery of the SMS. iPLATO will be responsible for giving each participant a unique study identifier. The document that links this unique code with the participants’ NHS number will be retained by iPLATO (and the GP practice) until the data has been analysed and will NOT be shared with the research team. The research team at NCL will only be provided with a fully pseudonymised dataset.

All electronic documents will be stored in a password protected folder on Newcastle University Teams accessible by only the research team. Source documents will include:

- Anonymised screening logs- (numbers of eligible patients and reasons why participants are ineligible)
- Pseudonymised electronic case report forms (eCRFs) with information about each individual patient participant.

## 11.3 Access to Data

- Only the core team members will have access to the data files and be able to manipulate the data.
- Direct access will be granted to authorised representatives from the Sponsor, host institution and the regulatory authorities to permit trial-related monitoring, audits and inspections.

11.4	Archiving

- The CI and RA will be responsible for archiving data upon completion of the study.
- Anonymised datasets and corresponding meta-data will be archived in the Newcastle University repository (data.ncl) for 10 years after the end of the study.

# 12. MONITORING, AUDIT & INSPECTION

- The day-to-day study conduct, and monitoring will be provided by BN and AG. They will maintain regular contact with the iPLATO team during the recruitment and allocation phases to provide support and record any issues that may arise.
- The PMG – consisting of the wider project team (see separate file on responsibilities) will monitor progress of the pilot and provide oversight and advice.
- The study may be subject to inspection and audit by Newcastle University.

# 13. ETHICAL AND REGULATORY CONSIDERATIONS

## 13.1 Research Ethics Committee (REC) review & reports

- Ethical approval from REC/HRA will be obtained prior to the commencement of the study.
- Substantial amendments will not be implemented until reviewed by NHS REC.
- Confidentiality Advisory Group approval will be sought to allow iPLATO to receive the identifiable data (DOB, NHS numbers) of patients without informed consent, (to enable them to send them text messages as part of the study). DPAs between the practices and iPLATO will be created to enable them to process this data.
- All correspondence with the REC will be retained.
- The CI will notify the REC of the end of the study.
- If the trial is ended prematurely, the CI will notify the REC, including the reasons for the premature termination
- Within one year after the end of the trial, the CI will submit a final report with the results, including any publications/abstracts, to the REC

## 13.2 Peer review

This study has been reviewed by an Oversight Group (Department of Health and Social Care and Office for Health Improvement and Disparities) and Scientific Advisory Board (independent experts from the field of health and social care research). Both of these groups provide oversight and advice to the unit (National Institute of Health and Social Care Research Policy Research Unit in Behavioural Science) who are conducting this research. It has also undergone internal review by the Unit’s senior investigators (Prof Angel Chater and Prof Mike Kelly) who are not directly involved in the project.

## 13.3 Public and Patient Involvement

### 13.3.1 Design of research

People with SMI, and who care for people with SMI were consulted in the early stages of the study design. They support the study, believe it important. They said the animation and leaflet were helpful and links should be included in the text reminder (webpage less preferable). The group also provided input into the text to include in the reminder- making it more inclusive and easy to read than originally proposed.

Concerns raise were about the whether SMS reminder may exclude some people who might not have data to connect to the internet to watch the animation or download the leaflet. There was a concern raised that some people may not open the links in the text message as they are wary that it may contain a virus or be some sort of scam. This pilot trial will measure both the number of women who receive the text and that click on the links within the text to provide further insights about this potential barrier.

PPI reps could not foresee any harm/distress being caused by the text reminder beyond what a usual letter or appointment would cause. However, it was noted that for some even the thought/conversation of CS may trigger further anxiety in women with bad past experiences of CS. To reduce the risk of anxiety that and information sheet about the study and invitation may cause, we will not contact eligible participants directly about the study, rather we have sought CAG approval to collect pseudonymised confidential sensitive data without consent.

### 13.3.2 Management and dissemination of research

We have a PPI rep (carer of a woman with SMI) who is part of our trial management team who will attend meetings and input into the management and conduct of the research for its duration. She will also help with the dissemination of the findings of the study.

## 13.4 Regulatory Compliance

- Before any site can enrol patients into the study, the CI will ensure that appropriate approvals from participating organisations are in place.
- For any amendment to the study, the CI in agreement with the sponsor will submit information to the appropriate body in order for them to issue approval for the amendment.
- The CI or designee will work with sites (study delivery team) so they can put the necessary arrangements in place to implement the amendment to confirm their support for the study as [amended](http://www.hra.nhs.uk/resources/after-you-apply/amendments/).

## 13.5 Protocol compliance

- Accidental protocol deviations will be documented on relevant forms and reported to the CI and Sponsor immediately.
- The research team will speak with participating practices and iPLATO at the end of the study to determine whether the protocol was workable and what might need to change for a future full trial

## 13.6 Notification of Serious Breaches to GCP and/or the protocol

- A “serious breach” is a breach which is likely to effect to a significant degree –
  (a) the safety or physical or mental integrity of the participants of the trial; or
  (b) the scientific value of the trial
- the sponsor will be notified immediately of any case where the above definition applies during the trial conduct phase

## 13.7 Data protection and patient confidentiality

All investigators and trial site staff will comply with the requirements of the Data Protection Act 1998 GDPR 2018 with regards to the collection, storage, processing and disclosure of personal information and will uphold the Act’s core principles.

(See data management plan)

## 13.8 Financial and other competing interests for the chief investigator at each site and committee members for the overall trial management

- None of the investigators or committee members have any conflict of interest to declare.

## 13.9 Indemnity

- NHS indemnity covers NHS staff involved in the trial (GPs and practice managers) for potential liability in respect of negligent harm arising from the conduct of the study.
- Newcastle University have provided indemnity insurance for the management and design of the study.
- IPLATO have provided indemnity insurance for the conduct of the study

## 13.10 Amendments

- If there is a need to make an amendment to the protocol (for example, change in CI, study documentation) the CI will complete the notification of tool and send it to the study sponsor. This form will indicate whether it is a substantial or non-substantial amendment. It is the sponsors responsibility to decide whether an amendment is substantial or non-substantial for the purposes of submission to the MHRA and/or REC for review and approval.
- If it is a substantial amendment, the sponsor must submit a valid notice of amendment to the REC for consideration. The REC will provide a response regarding the amendment within 35 days of receipt of the notice.
- The CI and study sponsor will be responsible for notifying NHS England and Improvement (the host NHS organisation in England) and trial sites (GP practices) of any amendments via email using the [template emails](https://www.hra.nhs.uk/approvals-amendments/amending-approval/) provided by the HRA.
- The amendment history will be tracked by ensuring that the title of the documentation are saved with the version and date each time.

## 13.11 Access to the final trial dataset

- Only the CI and co-investigators involved in data analyses (BN, AB, LV) will have access to the full dataset. This will help to ensure that the overall results are not disclosed prior to the main publication. Site investigators (GP collaborators) will not have access to the full dataset unless a formal request describing their plans is approved by the TMG.

# 14. 14 DISSEMINATION POLICY

## 14.1 Dissemination policy

- The trial protocol will be made publicly available upon the Open Science Framework upon HRA approval having been obtained.
- Upon completion of the trial, the data will be analysed and tabulated and a manuscript prepared which will published in open access journals
- DHSC will have 28 days to review publications before they are published.
- NIHR will be acknowledged within the publications
- Patient representatives and participating practices involved in the study will receive a short summary of the research findings. Practices are welcome to share this with the individuals who participated (as the trial team will not have names and addresses) and/or post the summary on their websites.

## 14.2 Authorship eligibility guidelines and any intended use of professional writers

- Authorship of publications will be based on the criteria outlined by the [International Committee of Medical Journal Editors.](https://www.icmje.org/recommendations/browse/roles-and-responsibilities/defining-the-role-of-authors-and-contributors.html)
- It is anticipated that FG will be the lead author on most outputs from this study.

# 15. REFERENCES

1. [*https://www.gov.uk/government/publications/severe-mental-illness-inequalities-in-cancer-screening-uptake/severe-mental-illness-smi-inequalities-in-cancer-screening-uptake-report*](https://www.gov.uk/government/publications/severe-mental-illness-inequalities-in-cancer-screening-uptake/severe-mental-illness-smi-inequalities-in-cancer-screening-uptake-report)
2. <https://www.gov.uk/government/publications/cervical-screening-call-and-recall-administration-best-practice/cervical-screening-call-and-recall>
3. Office for Health Improvement & Disparities. Premature Mortality in Adults with Severe Mental Illness (SMI); 2023. https://www.gov.uk/government/publications/premature-mortality-in-adults-with-severe-mental-illness/premature-mortality-in-adults-with-severe-mental-illness-smi).
4. Tuschick, E., Barker, J., Giles, E. L., Jones, S., Hogg, J., Kanmodi, K. K., Sill, J., & Sykes, K. (2024). Barriers and facilitators for people with severe mental illness accessing cancer screening: A systematic review. Psycho-oncology, 33(1), e6274. https://doi.org/10.1002/pon.6274)
5. Office for National Statistics. Cancer registration statistics, England: 2017 - Cancer diagnoses and age-standardised incidence rates for all types of cancer by age, sex and region including breast, prostate, lung and colorectal cancer; 2019. https://www.ons.gov.uk/peoplepopulationandcommunity/healthandsocialcare/conditionsanddiseases/bulletins/cancerregistrationstatisticsengland/2017
6. Kerrison, R.S., Jones, A., Peng, J. et al. Inequalities in cancer screening participation between adults with and without severe mental illness: results from a cross-sectional analysis of primary care data on English Screening Programmes. Br J Cancer 129, 81–93 (2023). https://doi.org/10.1038/s41416-023-02249-3
7. Hu, K., Wang, J., Sparén, P., Herweijer, E., Sjölander, A., Adami, H. O., Valdimarsdóttir, U., Sundström, K., & Fang, F. (2023). Invasive cervical cancer, precancerous lesions, and cervical screening participation among women with mental illness in Sweden: a population-based observational study. The Lancet. Public health, 8(4), e266–e275. <https://doi.org/10.1016/S2468-2667(23)00026-9>
8. Martens, P. J., Chochinov, H. M., Prior, H. J., Fransoo, R., Burland, E., & Need To Know Team (2009). Are cervical cancer screening rates different for women with schizophrenia? A Manitoba population-based study. Schizophrenia research, 113(1), 101–106. https://doi.org/10.1016/j.schres.2009.04.015
9. Murphy, K. A., Daumit, G. L., McGinty, E. E., Stone, E. M., & Kennedy-Hendricks, A. (2021). Predictors of cancer screening among Black and White Maryland Medicaid enrollees with serious mental illness. Psycho-oncology, 30(12), 2092–2098. https://doi.org/10.1002/pon.5815
10. Marrie, R. A., Walld, R., Bolton, J. M., Sareen, J., Patten, S. B., Singer, A., Lix, L. M., Hitchon, C. A., Marriott, J. J., El-Gabalawy, R., Katz, A., Fisk, J. D., Bernstein, C. N., & CIHR Team in Defining the Burden and Managing the Effects of Psychiatric Comorbidity in Chronic Immunoinflammatory Disease (2021). Effect of comorbid mood and anxiety disorders on breast and cervical cancer screening in immune-mediated inflammatory disease. PloS one, 16(8), e0249809. <https://doi.org/10.1371/journal.pone.0249809>
11. Public Health England. Guidance. Cervical screening: programme overview; 2021. https://www.gov.uk/guidance/cervical-screening-programme-overview
12. Launders (2020) Relative Incidence and Mortality Rates of Common Cancers in People with Severe Mental Illness in UK Primary Care file://ad.ucl.ac.uk/homev/rgjudav/Documents/Amainfolders/academic%20general%20incl%20walport/2022/SSRN-id3582847.pdf
13. Huf 2020 Behavioral economics informed message content in text message reminders to improve cervical screening participation: Two pragmatic randomized controlled trials <https://www.sciencedirect.com/science/article/abs/pii/S0091743520301948?via%3Dihub>
14. Lamontagne F (2021) The development, usability and acceptability of a cervical screening informed-choice tool for women with severe mental illness. PhD Thesis. http://repository.uwl.ac.uk/id/eprint/7787/1/Lamontagne-Godwin%20-%20PhD%20Thesis%20(Feb%2021%20-%20FINAL).pdf
15. PHE 2022 https://www.gov.uk/government/publications/cervical-screening-support-for-people-who-find-it-hard-to-attend/cervical-screening-support-for-people-who-feel-anxious-about-attending
16. [*https://www.jostrust.org.uk/shop/cervical-screening-and-cervical-abnormalities/information-booklet-support*](https://www.jostrust.org.uk/shop/cervical-screening-and-cervical-abnormalities/information-booklet-support)
17. [*https://www.youtube.com/watch?v=75MUQVwv908*](https://www.youtube.com/watch?v=75MUQVwv908)
18. L[awrenson, J.](https://openaccess.city.ac.uk/view/creators_id/j=2Eg=2Elawrenson.html) Graham-Rowe, E., [Lorencatto, F.](https://openaccess.city.ac.uk/view/creators_id/fabiana=2Elorencatto=2E2.html) , Rice, S., Bunce, C. V., Francis, J., Burr, J. M., Aluko, P., Vale, L. D., Peto, T., Presseau, J., Ivers, N. M. & Grimshaw, J. M.(2018). What works to increase attendance for diabetic retinopathy screening? An evidence synthesis and economic analysis. Health Technology Assessment, 22(29), doi: [10.3310/hta22290](https://doi.org/10.3310/hta22290)

# 16. APPENDICIES

# 17. Appendix 1 – Amendment History

| Amendment Number | Protocol version no. | Date issued | Author(s) of changes | Details of changes made |
| --- | --- | --- | --- | --- |
| 1 | V2 15 March 23 | 13^th^ Sept 23 | JL | Non-substantial:  This is a pilot study of an enhanced text message reminder for cervical screening. The enhancement is a hyperlink, in the text message reminder, to information designed by and for people who are anxious about cervical screening. This information is publically available on the NHS National Screening website. We have been made aware that NHS England are including a link to this information in their paper invitation letter sent to people who are due to be screened. This means those approached to participate will see the link to the information in the invitation letter from the National Screening Service. However, this is not a hyperlink and requires people to type in the address manually (rather than click on it within a text message). As there is the potential for control group participants to have accessed the information we would like to ask them when they attend for screening whether they did so. This would be a short on-line questionnaire completed by the practice screening staff.  Participant procedure: Control group participants who attend for screening will be asked if they accessed the information from the link in the NHS National Screening Service invitation letter. If they did access the information they will be asked if it was helpful. The questionnaire will be on-line and administered by the person conducting the screening test.  Other documentation: A short on-line questionnaire has been designed specifically for control participants. |
| 2 | V3 28^th^ Feb 24 | Submitted: 28^th^ Feb 24 | FG | Substantial:  We have changed the protocol to reflect an update in the CI on the study and we've added the NHS REC number. We've updated the study design in how participants are approached about the study. We are applying for CAG approval to enable us to have GP practices consent on behalf of participants to collect data participant data as part of the study. Participants will be notified of the study and able to opt-out of their data being used via a link at the end of the SMS reminder messages. We've also updated the study design to reflect that iPLATO will no longer collect participant data remotely from GP records. iPLATO will now only facilitate the delivery of the text messages to eligible patients. To do this practice staff will share with iPLATO the date of birth and NHS number patients they have identified as eligible. iPLATO will then randomly allocated eligible patients to receive either the control or enhanced SMS. iPLATO will assign each participant a PID number and provide practices with that PID number. 18 weeks after the SMSs have been sent, practice staff will collect patient demographic and health information from GP records. Practice staff will then remove identifiable data from the dataset and share it with the research team at NCL conducting the data analysis. A further additional change is that initally participants who attend for screening were going to be asked if they accessed the information from the link in the NHS National Screening Service invitation letter/ SMS text message reminder. The questionnaire was going to be on-line and administered by the person conducting the screening test. Now this questionnaire is going to be sent directly to participants via SMS 18 weeks after the SMS reminder. iPLATO will collate and pseudonymise (with the PID) any survey responses received and share with the research team for analysis. |

{Enter all amendments to the protocol here whether substantial or non-substantial. Substantial amendments will require approval by the NHS REC. Non-substantial amendments should be sent to the NHS REC for acknowledgement only}

# 18. Appendix 2: Statistical Analysis Plan

OPTIMISE	Statistical Analysis Plan v1.0

PHSI Biostatistics Research Group

Statistical Analysis Plan (SAP) for the OPTIMISE Trial

Behavioural SMS reminders with link to decision tool to improve uptake of cervical screening amongst people with severe mental illness that are overdue: a pilot randomised controlled trial

SAP Version number: 1.0

SAP date: 02 June 2025

**IRAS Number: 323832**

**NHS REC Reference: 23/WM/0037**

**Research Registry & References: ISRCTN12558681**

**RESEARCH Sponsor Name: The University of Newcastle Upon Tyne Foundation**

**Funder Name: National Institute for Health Research Policy Research Unit in Behavioural Science (project reference PR-PRU-1217-20501).**

**Funder Reference: 202223 23 Lecouturier**

**Trial Statistician: Dr Andrew Bryant**

**Signature**:	**Date**: 02/06/2025

**Chief investigator: Beth Nichol**

**Signature**: **Date**: 01/05/2025

Page **1** of **14**

OPTIMISE	Statistical Analysis Plan v1.0

**CONTENTS**

[1. INTRODUCTION	**3**](#_Toc25061)

[1.1 Trial Summary	3](#_Toc25062)

[1.2 Recall pathway diagram for cervical screening and the point at which patients become classed as non-responders	5](#_Toc25063)

[1.3 Flow chart of trial design	6](#_Toc25064)

[2. TIMING AND REPORTING INTERIM AND FINAL ANALYSES	**7**](#_Toc25065)

[2.1 Randomisation	7](#_Toc25066)

[2.2 Blinding	7](#_Toc25067)

[2.3 Follow-Up and Schedule of Events	7](#_Toc25068)

[Schedule of Events	8](#_Toc25069)

[3. STUDY POPULATION	**8**](#_Toc25070)

[3.1 Baseline Participant Characteristics	8](#_Toc25071)

[3.2 Defining Populations for Analysis	11](#_Toc25072)

[4. SAFETY ANALYSIS	**11**](#_Toc25073)

[5. OUTCOMES	**11**](#_Toc25074)

[5.1 Definition and Calculation of Outcome Measures	11](#_Toc25075)

[6. DATA ANALYSIS	**12**](#_Toc25076)

[6.1 Details of Sample size calculation	12](#_Toc25077)

[6.2 Missing data	12](#_Toc25078)

[6.3 Descriptive analyses	12](#_Toc25079)

[6.3.1 Participant characteristics at baseline	12](#_Toc25080)

[6.3.2 Outcome measures	13](#_Toc25081)

[6.4 Secondary analyses	13](#_Toc25082)

[6.5 Subgroup Analysis	13](#_Toc25083)

[6.6 Sensitivity Analyses	13](#_Toc25084)

[6.7 Statistical Software	13](#_Toc25085)

[7. STORAGE AND ARCHIVING	**14**](#_Toc25086)

[8. REFERENCES	**14**](#_Toc25087)

Page **2** of **14**

OPTIMISE	Statistical Analysis Plan v1.0

# 19. INTRODUCTION

This statistical analysis plan (SAP) provides guidelines for the data analysis to be undertaken for the presentation and publication of the OPTIMISE trial. This plan, along with all other documents relating to the analysis of this trial, will be stored in the ‘Statistical Documentation’ of the Trial Master File (TMF) and the final signed SAP will also be stored at a secure NUTH facility after trial end according to current Trust R&D arrangements and for a duration in line with research data ‘best practice’ management.

To avoid a large overlap and duplication of material from the trial protocol, the SAP will mostly restrict to information and analyses relevant to this document, but will provide short summaries in certain sections where appropriate. Please refer to the protocol for full details of the trial including the study population, aims and objectives, details of sample size (and calculation), power, recruitment and randomisation, as well as full descriptions of the interventions. Other aspects of the trial and its conduct such as measures of quality control are also provided in full detail in the protocol.

The overarching purpose of the trial is to assess the feasibility of participant identification and implementation processes in the trial.

## Trial Summary

| Trial Title | Behavioural SMS reminders with links to online information and support to improve uptake of cervical screening (CS) amongst people with severe mental illness (SMI) that are overdue: a pilot randomised controlled trial | |
| --- | --- | --- |
| Acronym | OPTIMISE | |
| Summary of Trial Design | Pilot and feasibility Individual randomised controlled trial with model based economic evaluation to identify the feasibility of and target difference for subsequent definitive RCT. | |
| Summary of Participant Population | All people with a cervix with SMI between 24- 64 years who are classed as non-responders (have not had a CS during the age-appropriate interval i.e. within the previous 3.5 years for patients aged 24-49 within the previous 5.5 years for patients aged 50-64). | |
| Planned Sample Size | 88 (44 per arm). | |
| Planned Number of Sites | Up to 30 GP practices across North and South East London | |
| Intervention Duration | One SMS reminder | |
| Follow Up Duration | 18 weeks | |
| Planned Trial Period | November 2024 to June 2025 |  |
|  | Objectives | Outcome Measures |
| Primary | To assess the feasibility of participant identification and implementation processes | Feasibility measures: number of eligible patients, number excluded in screening and reasons for exclusion, Number of potential participants that dissent/opt-out of the study, Number of participants for which mobile number is missing/invalid, number of texts |

Page **3** of **14**

|  |  | delivered, number of times links opened. |
| --- | --- | --- |
| Secondary | To rehearse data collection and data analyses for effectiveness and economic evaluation and refine for  subsequent definitive trial    Estimate the minimum level of additional effectiveness of enhanced SMS cervical screening reminders  that would be cost-effective      iPLATO to collect basic information on whether intervention participants who attend for screening accessed the intervention information. | CS status of each participant 18week post intervention (CS  appointment attended)      Costs of enhanced and usual text messages (including delivery of the SMS messages and time and resources required from GP practices)    GP resource use/research activity |

.

OPTIMISE	Statistical Analysis Plan v1.0

## Recall pathway diagram for cervical screening and the point at which patients become classed as non-responders.

PNL-Prior Notification List, NTTD- Next Test Due Date Cervical screening: guidance for call and recall administration best practice - GOV.UK (www.gov.uk)

## Flow chart of trial design

Pilot randomised controlled trial (parallel design) to rehearse and test processes for a fullscale trial.

**Quantitative data analyses**

:

•

Screening rates % (eligible, n/ screened)

•

Number of patients that opt

-

out (n)

•

Numbers that dissent

Numbers that did not receive allocated intervention/ SMS delivery status

•

Uptake at 18 weeks (attendance at CS appointment)

•

Whether intervention participants who attend for screening

accessed the information

To intervention (n= )

Patients receive text reminder with link to

webpage and animation

Patients screened for eligibility by GP (n=

)

Excluded (n=)

Reasons (…)

Not meeting inclusion

criteria (n=)

Randomised (n= )

To control (n= )

Patients receive usual text reminder

Patients identified in computer search

by practice staff (n=)

GP practices provide iPLATO a list of

eligible patients

-

people with SMI overdue

cervical screening (non

-

responders).

iPLATO then randomly allocate patients to

each arm and assigns PID numbers.

At 18 weeks, practice staff run search of GP records for participant health information and whether an CS

appointment has been attended for screening (Y/N). All participants are sent a link to a survey (the exact

survey depends on whether they are assigned

to the intervention or control group). Practice staff

complete GP research activity proforma.

**Inclusion criteria**

•

Aged between 24

-

64

years

•

Diagnosed with SMI

•

Classed as non

-

responders on

CSAS list sent to GP practices.

•

Registered with a participating

practice

**Exclusion criteria**

•

Those the general practitioner

considers it inappropriate to

approach.

•

Those it is considered

inappropriate to approach

•

Ineligible for screening

•

Those that have dissented to

have their data used for

research purposes (Type 2 opt

-

outs).

•

Those that have dissented to

receive SMS text

messages

**Health economic analysis**

:

Cost of enhanced SMS reminder

compare with usual (proforma),

time taken for practices to

identify non

-

responders with

SMI and other research

activities (proforma)

Screened

Assessment

# 20. TIMING AND REPORTING INTERIM AND FINAL ANALYSES

This is a multisite trial involving GP practices within primary care networks

(PCNs)/federations across North and South East London where SMI prevalence is relatively high compared to the national average.

There will be no planned interim analyses carried out as this is a pilot and feasibility trial. Therefore, no independent Data Monitoring and ethics Committee (DMC) will be convened to undertake any independent reviews at this stage of the trial.

## Randomisation

The randomisation process is outlined in the protocol. In summary, iPLATO will be used to generate random numbers to use to randomise the participants in the trial. Participants will be allocated using 1:1 randomisation.

## Blinding

Participants and GP staff will be blinded to the group allocation.

## Follow-Up and Schedule of Events

The trial will end when the data for the last participant is retrieved from GP records by practice staff and the survey data has been collected from iPLATO.

To assess the feasibility of general practice resource use, practice managers will be consulted about resources required to undertake the various research activities as part of this study including: identifying eligible participants (complete detailed screening logs), collecting information about participants through searches of GP records at 18 weeks, and pseudonymising and sharing data collected with the research team at NCL. Details of these conversations will be recorded on a proforma completed by the research team at NCL.

iPLATO will randomise and send those in the intervention arm the enhanced text message reminder and those in the control group the standard reminder. After 18 weeks, GP staff will run a search of their clinical systems and complete a case report form for each study participant collecting the following information:

- patient demographics data (e.g., age, gender, ethnicity, first part of postcode to derive IMD)
- patient characteristics (e.g., year of SMI diagnosis, comorbidities, date of last screening appointment
- Screening appointment attended following SMS reminder(Y/N)

This data together with allocation status (control/intervention) will be pseudonymised and shared with the data analysis team at Newcastle University (each participant will have a PID rather than a personal identifier). The PID key will be retained by iPLATO and the participating practice and destroyed once the data has been analysed.

## Schedule of Events

1. NCL provides participating GP practices with search terms/code to identify patients with SMI that are non-responders.
2. GPs review the list to determine whether patients are eligible to participate in the study and it is appropriate to include them
3. GP practices provide iPLATO with a list of eligible patients (NHS numbers, Date of Birth) with SMI that are overdue cervical screening (non-responders)
4. iPLATO randomises patients to receive either a control text reminder (no link) or intervention enhanced text reminder (with link). (They use the NHS number to access clinical systems to send the text message). At the end of the text message there will be a link to a GP privacy notice and a link to enable them opt-out of this specific study.
5. iPLATO will receive and remove any study specific opt-outs from the list of participants.
6. iPLATO will collect SMS feasibility measures and save with DOB, NHS and PID numbers on a participant case report form and securely share it with GP practices.
7. After 18 weeks, iPLATO will send all participants a SMS with link to a survey to ask whether they accessed the text from their invitation letter (both control and intervention groups) information materials that are the focus of this study (intervention group only), which ones (animation and/or textual, intervention group only) and whether they watched/read the information. iPLATO will receive survey responses and share with the data analysis team at Newcastle University.
8. After 18 weeks, practice staff will search the GP systems for the agreed patient data and add it to the participant case report form.
9. They will remove the NHS numbers and DOBs from the participant case report form and securely share the pseudonymised dataset with the team at NCL.
10. GP staff will provide the data analysis team at Newcastle University with pseudonymised dataset.
11. GP staff and iPLATO will be consulted regarding the amount of time they have spent on the research activities as part of the economic and feasibility assessment of this study.

# 21. STUDY POPULATION

## Baseline Participant Characteristics

All people with a cervix with SMI between 24- 64 years that are classed as non-responders (have not had a CS during the age-appropriate interval i.e. within the previous 3.5 years for patients aged 24-49 within the previous 5.5 years for patients aged 50-64) and are deemed eligible and appropriate to include.

Demographic, clinical and baseline characteristics (e.g. age will be summarised descriptively across treatment groups and overall).

Significance testing to compare baseline characteristics will not be carried out in the study.

The following routinely collected information and clinical assessments will be collected:

• Demographics o Age

o Ethnicity o Index of Multiple Deprivation

- Year of SMI diagnosis
- At least one long term condition on the QoF disease register (Y/N)
- Date of last screening appointment
- Screening status during intervention period (Y/N)

**Table 1: Comparison of participant characteristics at baseline between intervention and control group**

|  | **Intervention group (n= )** | **Control group (n= )** |
| --- | --- | --- |
| **Age (years) at randomisation:** |  |  |
| Mean (SD) |  |  |
| Median (IQR) |  |  |
| Range: Min-Max |  |  |
| **Ethnicity** |  |  |
| White |  |  |
| Black, African, Caribbean, or Black British |  |  |
| Mixed or Multiple ethnic groups |  |  |
| Asian or Asian British |  |  |
| Other |  |  |
| **Index of Multiple Deprivation** |  |  |
| 10% (in top 10% deprived area) |  |  |
| 20% |  |  |
| 30% |  |  |
| 40% or less in deprived area |  |  |
| **Years since SMI diagnosis** |  |  |
| Mean (SD) |  |  |
| Median (IQR) |  |  |
| Range: Min-Max |  |  |
| **At least one long term condition on the QoF disease register** |  |  |
| Yes |  |  |
| No |  |  |
| **Time from date of last screening appointment to randomisation** |  |  |
| Mean (SD) |  |  |
| Median (IQR) |  |  |
| Range: Min-Max |  |  |
| **Screening status during intervention period (Y/N)** |  |  |
| Yes |  |  |
| No |  |  |

## Defining Populations for Analysis

Any participant randomised into the trial, regardless of whether they received intervention or not, will be included in the descriptive analyses. Definitive trial would follow an intention to treat analysis.

# 22. SAFETY ANALYSIS

Safety reporting is not applicable to this trial.

# 23. OUTCOMES

## Definition and Calculation of Outcome Measures

Feasibility parameters (screening rates, recruitment rates, missing data) will be presented in simple descriptive tables presenting percentages, means and standard deviations for each arm of the study.

Where applicable, full details about how outcomes have been calculated (as well as dealing with missing data and any recoding) for each primary and secondary outcome are given below.

**Primary feasibility outcomes**

All calculations will be carried out on completion of data collection.

i.	Description of screening results–

a. % eligible of screened within the trial period for each arm ii.	Table or graph to show recruitment by month and GP practice against target

iii. Data will be summarised as the number and percentages of eligible participants who were recruited as well as:
     a. % of those randomised (number of participants randomised/total number eligible)
     b. % who opted out during the trial period [1-(number of participants followedup at the end of the trial/total number randomised)]
     c. Number and % of uptake for a cervical smear (Number who had a smear test/total number of participants followed-up)

The above will be displayed in the form of a CONSORT diagram –See dummy Figure in Section 1.3 above.

iv. Descriptive statistical analysis of primary outcome variables.

The results will be presented using tables reporting data at baseline and 18 weeks follow-up.

**Pilot effectiveness (secondary) outcome measures**

We will assess CS status of each participant 18-week post intervention (CS appointment attended).

GP resource use/research activity will be explored by using the iPLATO to collect basic information on whether intervention participants who attend for screening, actually accessed the intervention information.

Costs of enhanced and usual text messages and cost effectiveness outcomes will be analysed by the health economist on the project.

For categorical variables (e.g. index of multiple deprivation), we will calculate the number of participants in each arm in each category of interest and the number of participants assessed at the appropriate timepoint, in order to compute percentages in each arm.

For discrete interval and continuous outcomes (e.g. age), we will calculate the mean and standard deviation of the outcome of interest in each arm and the number of participants assessed in each arm and time-point. To investigate the shape of the distributions we will also report the 5-number summary (median, IQR and range) in each arm.

We will not impute missing outcome data for any of the outcomes.

# 24. DATA ANALYSIS

As this is a pilot trial, analyses will be descriptive in nature and at the participant level.

## Details of Sample size calculation

The proposed sample size in the trial and further details are documented in the protocol.

## Missing data

Implementation of the trial as intended, and completeness of outcomes will be recorded as the main feasibility outcomes in the trial. Data completeness and progress reports may be generated and reviewed at TMG meetings.

We will not consider the use of any imputation methods since this is a pilot and feasibility trial.

## Descriptive analyses

Descriptive analysis of the study population will be summarised for each randomised group and will include:

- Baseline participant characteristics (section 3.1)
- Feasibility outcomes (section 6.3.2.1)
- Pilot effectiveness outcomes (section 6.4)

A full description of outcomes that will be reported in the trial is given in section 5.1.

### Participant characteristics at baseline

Baseline (BL) characteristics of the study population will be summarised separately within each randomised group. Characteristics such as ethnicity will be summarised by reporting the number (%) in each category, whereas covariates such as age will be reported as mean (SD) and range or median (IQR) as appropriate (see dummy Table 1).

### Outcome measures

This trial is a pilot and feasibility trial. The main analyses will focus on feasibility outcomes and their completeness. Descriptive measures for all variables will be given; absolute and relative frequencies for categorical data, median, minimum and maximum for continuous data.

#### Feasibility outcomes

**Primary endpoint:**

**Interventional group versus control**

|  | Interventional group  (n= ) | Control group (n= ) |
| --- | --- | --- |
| Screening: Number (%) |  |  |
| Eligible: Number (%) |  |  |
| Recruitment: Number (%) |  |  |
| Randomised: Number (%) |  |  |
| Uptake of smear: Number (%) |  |  |
| Mobile invalid or missing: Number (%) |  |  |
| Texts delivered: Mean (SD)[Range] |  |  |
| Animation link opened (%) |  |  |
| Webpage link opened (%) |  |  |

## Secondary analyses

See Section 6.3.3 for details about the primary and secondary outcomes.

There will be no formal hypothesis testing as this is just a pilot and feasibility trial. However, analyses of the primary outcome measure that would be conducted in a definitive trial will be rehearsed to look for trends in the data. Exploratory logistic regression will be performed to assess the association between trial arms and uptake before and after adjusting for specified baseline characteristics (age (continuous) and ethnicity (categorical)). Absolute rates of uptake will be reported. This analysis will not be considered definitive and will be interpreted in a cautious manner however will provide effect sizes to be applied in a future fully powered trial.

For this dichotomous (binary) outcome, we will extract the number of participants in each intervention arm who went for their cervical smear and the number of participants who were in the trial at 18 weeks follow-up and report percentages in each arm.

These analyses will be performed using the statistical package Stata (StataCorp, 2025).

## Subgroup Analysis

No subgroup analysis are planned.

## Sensitivity Analyses

No *a priori* sensitivity analyses will be conducted.

## Statistical Software

All trial data will be securely stored on the University’s Filestore Service, also known as Research Data Warehouse. The data will be accessible only to authorised members of the project team involved in the analysis.

Data will be transformed into Stata format or directly imported into Stata (depending on its format) and analyses will be carried out by the Trial Statistician downloading snapshots of the data at time-points agreed by the TMG. Software versions will be recorded at the time of analysis.

# 25. STORAGE AND ARCHIVING

iPLATO will access names and addresses of eligible participants only for the purpose and period of recruitment within each practice. Pseudonymised data will be transferred by iPLATO using the university encrypted drop off service. iPlATO will hold this data for 30 days only.

On recipe to of data from GP practices (pseudo anonymised patient data) and iPLATO (survey data), data will be securely stored on the University’s Filestore Service, also known as Research Data Warehouse. The data will be accessible only to authorised members of the project team involved in the analysis. This service is backed up daily with a highly available offsite mirror.

Data will be managed in accordance with best practice in the field and will comply with the

Data Protection Act and the University's own Information Security and Data Protection Policies [https://services.ncl.ac.uk/itservice/policies/]. Data will be saved and kept there for up to 3 months after the study has ended (September 2024 at the latest).

# 26. REFERENCES

StataCorp. 2025. Stata Statistical Software: Release 19. College Station, TX: StataCorp LLC.

1. SMI defined as: all people with a record indicating a diagnosis of schizophrenia, bipolar affective disorder or other psychoses, as well as those on lithium therapy, were identified within the sample data’s active cohort as ‘with SMI’. This definition is aligned with the definition used to construct the Quality and Outcome Framework (QoF) SMI register.
