# Supplementary material for: Behaviourally informed text message reminders to increase cervical screening attendance in people with severe mental illness: the OPTIMISE pilot randomised controlled trial
Source: BMJ Open. 2026 Sep 11;16(9):e122934. doi: 10.1136/bmjopen-2026-122934 (PMC13583816; doi:10.1136/bmjopen-2026-122934)
Supplement: Supplementary file 4 [file bmjopen-16-9-s003.docx]

**Supplementary Material 3:** Summary of findings from the rehearsal of the collection of data that would be required for an economic evaluation in a definitive trial.

SMS messages at iPLATO are costed based on credits (which cost 2.5p each), which have a character limit. Thus, the control SMS cost 5p (2 credits) each to send and the intervention cost 7.5p (3 credits) to send. Thus, the additional cost of the intervention was 2.5p per person. iPLATO reported spending an estimate of 244 hours on their involvement in the trial; mostly on programme delivery (53.3%), followed by programme preparation (34.4%) and external communications (12.3%), including with GP practices. Six of the 10 participating practices who returned outcome data provided feedback on time spent on the trial. GP practices spent an estimated average of 246 minutes in total on their involvement in the study (ranging from 110 to 420 minutes). Broken down by task, GP practice spent an average of 38 minutes on the admin for the study (ranging from 20 to 60 minutes), 49 minutes on searching for eligible patients (ranging from 20 to 130 minutes), and 158 minutes on extracting participant data (ranging from 60 to 260 minutes). Roles of each actor were also collected, allowing the costs to be calculated within a definitive trial by using a primary care resources calculation tool.
